# Identification of oleic acid as an endogenous ligand of GPR3

**DOI:** 10.1038/s41422-024-00932-5

**Published:** 2024-01-29

**Authors:** Yangjie Xiong, Zhenmei Xu, Xinzhi Li, Yuqin Wang, Jing Zhao, Na Wang, Yaning Duan, Ruixue Xia, Zhengbin Han, Yu Qian, Jiale Liang, Anqi Zhang, Changyou Guo, Asuka Inoue, Yu Xia, Zheng Chen, Yuanzheng He

**Affiliations:** 1 HIT Center for Life Sciences, School of Life Science and Technology, Harbin Institute of Technology, Harbin, Heilongjiang China; 2 MOE Key Laboratory of Bioorganic Phosphorus Chemistry & Chemical Biology, Department of Chemistry, Tsinghua University, Beijing, China; 3 Graduate School of Pharmaceutical Sciences, Tohoku University, 6-3, Aoba, Aramaki, Aoba-ku, Sendai, Miyagi Japan; 4 Frontiers Science Center for Matter Behave in Space Environment, Harbin Institute of Technology, Harbin, Heilongjiang China

**Keywords:** Cryoelectron microscopy, Hormone receptors

## Abstract

Although GPR3 plays pivotal roles in both the nervous system and metabolic processes, such as cold-induced thermogenesis, its endogenous ligand remains elusive. Here, by combining structural approach (including cryo-electron microscopy), mass spectrometry analysis, and functional studies, we identify oleic acid (OA) as an endogenous ligand of GPR3. Our study reveals a hydrophobic tunnel within GPR3 that connects the extracellular side of the receptor to the middle of plasma membrane, enabling fatty acids to readily engage the receptor. Functional studies demonstrate that OA triggers downstream G_s_ signaling, whereas lysophospholipids fail to activate the receptor. Moreover, our research reveals that cold stimulation induces the secretion of OA in mice, subsequently activating G_s_/cAMP/PKA signaling in brown adipose tissue. Notably, brown adipose tissues from *Gpr3* knockout mice do not respond to OA during cold stimulation, reinforcing the significance of GPR3 in this process. Finally, we propose a “born to be activated and cold to enhance” model for GPR3 activation. Our study provides a starting framework for the understanding of GPR3 signaling in cold-stimulated thermogenesis.

## Introduction

G-protein-coupled receptor 3 (GPR3) was an orphan receptor of class A G-protein-coupled receptors (GPCRs), and it is closely related to GPR6 and GPR12, all of which have a high level of constitutive G_s_ coupling activity.^1,2^ Initially, sphingosine-1-phosphate (S1P) and other lysophospholipids were proposed as ligands of GPR3, GPR6 and GPR12 in ligand screening assays using HEK293 cells in charcoal-stripped serum medium.^3^ Later, diphenyleneiodonium chloride (DPI) was indicated as an agonist of GPR3 by a similar approach.^4^ More recently, the nonpsychoactive phytocannabinoid cannabidiol was found to be an inverse agonist for GPR3, GPR6 and GPR12.^1,5^ Nevertheless, none of these reports have presented definitive evidence to substantiate their claims, and some of them even conflict with one another or cannot be replicated.^1,6,7^ As a result, the ligand of GPR3 remains elusive.

GPR3 was found to be largely expressed in neurons of various brain regions^8^ and shown to have an anti-proliferative effect in cerebellar neuron,^9^ promote neurite outgrowth^10^ and be important for neuronal cell survival,^11^ indicating a key role in the nerve system. Furthermore, GPR3 has been identified as a mediator of amyloid beta (Aβ) production,^12^ a hallmark of Alzheimer’s disease. Overexpression of GPR3 increases Aβ secretion, whereas ablation of GPR3 decreases Aβ production. In addition, GPR3 has been implicated in modulating emotional behavior,^7^ playing a role in neuropathic pain,^13^ and influencing addiction.^14^

Beyond its involvement in the nervous system, GPR3 is also associated with energy consumption and obesity. Notably, a nearly 20-fold increase in GPR3 expression in interscapular brown adipose tissue (iBAT) was observed following in vivo cold exposure of wild-type (WT) mice, and *Gpr3* knockout (KO) mice failed to mount a thermogenic response.^15^ A recent study demonstrated that lipolysis drives GPR3 to induce adipose thermogenesis upon cold stimulation.^16^ The mRNA level of GPR3 in adipocytes soars upon cold stimulation via lipolytic signal, resulting in a continuous G_s_ coupling and cAMP production, followed by rapid energy expenditure and thermogenesis.^16^ These findings make GPR3 an attractive target for metabolic disorders such as obesity and diabetes.

The high level of constitutive G_s_ coupling activity of GPR3 remains a mystery. There are two potential explanations for this constitutive activity. One possibility is that GPR3 is a “born to be active” receptor, which implies that it possesses an active conformation immediately after translation from mRNA, allowing it to continually associate with G_s_ protein. This intrinsic activity is seen in several orphan GPCRs. For example, GPR52 utilizes its extracellular loop 2 (ECL2) as a built-in ligand to activate itself,^17^ and GPR20 uses its N-terminal helix to activate the receptor.^18^ In the context of the GPR3 thermogenesis study, the researchers suggested that GPR3 might follow a similar mechanism for self-activation.^16^ In the meantime, these previous studies do not preclude the existence of an abundant supply of endogenous ligand within cells to promptly bind and activate GPR3 as soon as the receptor is translated from mRNA, in a term that we refer to as “born to be activated”.

Given the potential of GPR3 in neurodegenerative diseases and metabolic disorders, we sought to solve the structure of GPR3 in complex with G_s_ protein by cryo-electron microscopy (cryo-EM). Our study uncovers an endogenous ligand density in the ligand-binding pocket of GPR3. Combining mass spectrometry (MS) analysis and functional assays, we identify oleic acid (OA) (FA 18:1 n-9; n-9 means double bond at the 9th carbon position, C9) as a ligand of GPR3, and reveal key determinants of GPR3 ligand binding. Furthermore, we provide evidence that cold stimulates the secretion of OA to activate GPR3 in mice, and the activation is abolished when *Gpr3* gene is knocked out, filling the missing knowledge gap between cold-induced lipolysis and thermogenesis.

## Results

### The constitutive activity of GPR3

We first assessed the constitutive activity of GPR3 in a conventional GPCR reporter assay. The data showed that GPR3 exhibited a significantly high level of constitutive activity in the cAMP response element (CRE) reporter assay (G_s_ signaling), but not in the serum response element (SRE, G_i_ signaling), serum response factor response element (SRF-RE, G_12/13_ signaling) and nuclear factor of activated T-cell response element (NFAT-RE, G_q_ signaling) receptor assays^19^ (Supplementary information, Fig. S1a). This suggests that GPR3 exclusively activates G_s_ signaling pathway, aligning with the previous research.^1^ Next, we investigated whether GPR3 follows the self-tethering peptide activation model. This model has been observed in protease-activated receptors (PARs)^20,21^ and adhesion receptors (aGPCRs),^22^ where a cleaved peptide of the extracellular side of receptor dips back into the ligand-binding pocket of the receptor and activates it. To explore this, we conducted N-terminal deletions of GPR3 and assessed the activity of these truncated constructs through CRE reporter and cAMP assays. We observed that the N-terminal deletions of GPR3 (up to residue 36) did not diminish its constitutive G_s_ signaling of GPR3 (Supplementary information, Fig. S1b, c; protein expression level shown in Supplementary information, Fig. S4d). To explore the possibility of an endogenous ligand within the GPR3 pocket, we proceeded with structural determination.

### The overall architecture of GPR3/G_s_ complex

We expressed full-length GPR3 (Supplementary information, Fig. S1d) along with a Gα_s_ harboring two dominant negative mutations^23^ (G226A and A366S), as well as Gβ_1_ and Gγ_2_ in *Spodoptera frugiperda* (Sf9) insect cells (see Materials and Methods for details). We purified the proteins by a conventional membrane protein purification method of our lab^24^ and we included Nb35 during purification to facilitate formation of a stable GPR3/G_s_ complex^25^ (Supplementary information, Fig. S1e).

**Fig. 1:**
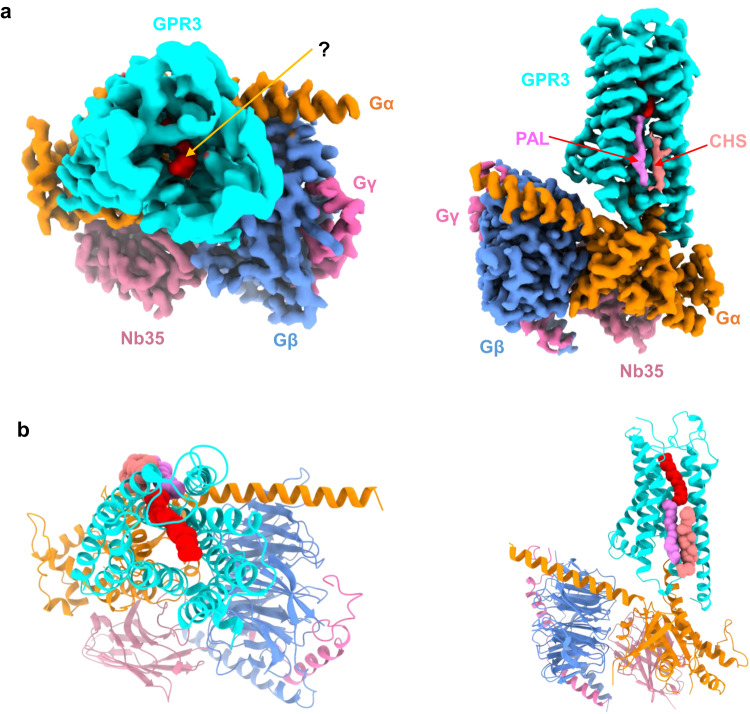
Overall structure of GPR3/G_s_ complex. **a** Orthogonal views of the cryo-EM density map of the GPR3/G_s_ complex. Left panel, top view; right panel, side view. “?” marks unknown ligand. PAL palmitic acid, CHS cholesteryl hemisuccinate. **b** Model of the complex in the same view and color scheme as shown in **a**.

Using cryo-EM single particle analysis, we solved the GPR3/G_s_ complex at a global resolution of 2.79 Å by the gold standard of Fourier Shell Correlation (FSC) = 0.143 criterion (Supplementary information, Fig. S2 and Table S1). The receptor side has a high-quality electron density map allowing unambiguous assignment of almost the whole receptor except the first 36 N-terminal residues, residues 232–240 of the intracellular loop 3 (ICL3) and residues after 311 at the C-terminus due to their high flexibility. The overall structure of GPR3/G_s_ complex resembles most GPCR/G-protein complexes in which the G-protein engages the receptor intracellularly via its Gα subunit (Fig. 1). Notably, the orthosteric ligand-binding pocket is widely open on the extracellular side with a slim worm-like unknown electron density visible from the top view (Figs. 1a, left panel and 2a). Additionally, two distinct lipid densities are present at the intracellular half of the transmembrane domain on a groove formed by transmembrane helices (TMs) 4, 3, and 5 (Fig. 1a, right panel; Supplementary information, Fig. S3a, b). Based on their resemblance to palmitic acid (PAL) and cholesteryl hemisuccinate (CHS) in a similar grooves of other class A GPCRs, for instance, cannabinoid receptor 2 (CB2; PDB: 6pt0),^26^ formyl peptide receptor 2 (FPR2; PDB: 6omm),^27^ growth hormone-releasing hormone receptor^28^ (GHRHR; PDB: 7cz5), and S1P receptor 1^29^ (S1PR1; PDB: 7evz), we assigned these densities to PAL and CHS, respectively. The key question is the identity of the unknown density in the orthosteric pocket (Figs. 1a, left panel and 2a).

### Identification of OA as a ligand of GPR3

**Fig. 2:**
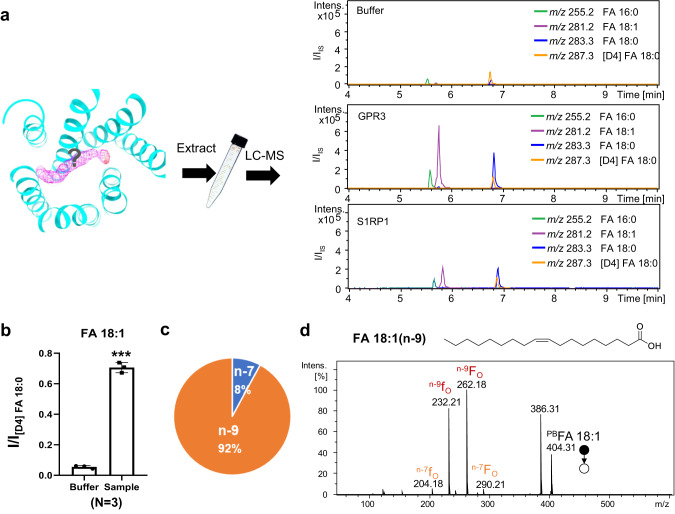
MS identifies OA as a ligand of GPR3. **a** Scheme of the MS approach for identification of OA as a ligand of GPR3. Extracted ion chromatogram of the detected FAs, including FA 16:0 (green), FA 18:1 (purple), FA 18:0 (blue), and [D4] FA 18:0 (orange). **b** Relative intensities of the detected FA 18:1 in sample and buffer. **c** Relative composition of C=C location isomers in FA 18:1. **d** 2-Acpy PB-MS/MS spectrum of FA 18:1. Upper panel, chemical structure of FA 18:1 (n-9).

**Fig. 3:**
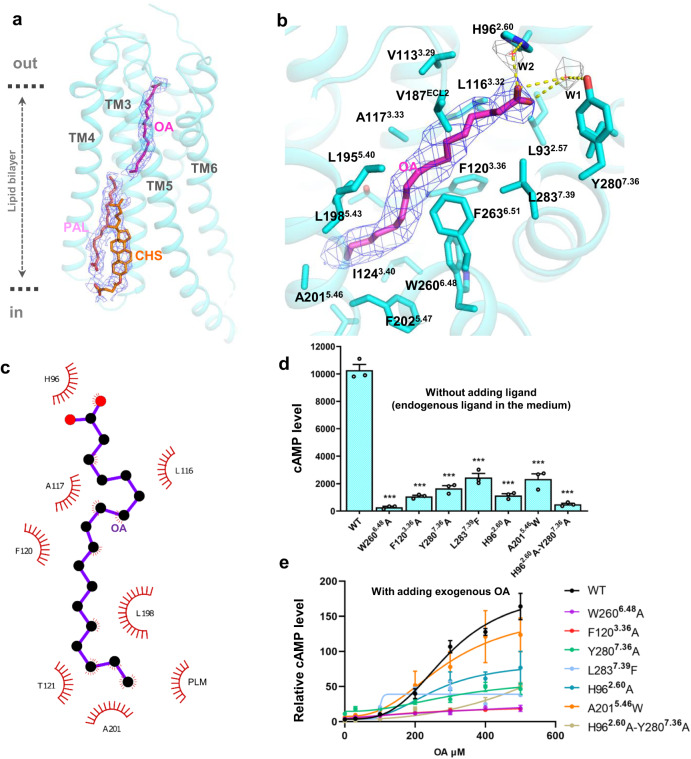
Ligand-binding pocket of GPR3. **a** The overall shape and density of 3 ligands of GPR3. Density maps of CHS, PAL and OA are set at contour levels of 3.0–5.0 in PyMol. **b** Detailed view of the ligand-binding pocket. Density maps of OA (blue mesh) and water molecules (W1 and W2, gray mesh) are set at contour levels of 5.0 and 3.0, respectively, in PyMol. **c** Analysis of the hydrophobic interaction between receptor and ligand via LigPlot. **d** cAMP assay of GPR3 mutants under condition without addition of exogenous ligand (endogenous ligand of the medium). Data are presented as means ± SEM; *n* = 3 independent samples; n.s. not significant; *P < 0.05; **P < 0.01; ***P < 0.001. Two-sided *t*-test. **e** OA dose-response curve of stably expressed GPR3 mutants. Data are presented as means ± SEM; *n* = 3 independent samples. OA was added to the cell culture medium at the designated dose.

We first examined whether this slim worm-like density corresponds to the N-terminal peptide of GPR3. The density of the unknown ligand is of high quality; however, we could not identify any side-chain information from the density map (Figs. 2a and 3b), ruling out the tethered peptide ligand theory. From structural viewpoint, the slim worm-like density is highly reminiscent of a lipid molecule. We therefore used MS to identify the lipid components of our GPR3/G_s_ complex. Lipids in the complex were extracted by a modified methyl tert-butyl ether method^30^ and subjected to liquid chromatography mass spectrometry (LC-MS) analysis. To ensure that the lipid signal observed in the LC-MS analysis is specifically from the GPR3 complex and is not due to the high levels of fatty acids (FAs) in the Sf9 cells used for complex expression, we conducted a parallel expression and purification of both the GPR3/G_s_ complex and the S1PR1/G_i_ complex. Specifically, the S1PR1/G_i_ and GPR3/G_s_ complexes were expressed in the same Sf9 cells at the same time and purified simultaneously. Subsequently, lipid extraction and LC-MS analysis were performed on both complexes at the same time, ensuring a direct and simultaneous comparison of their lipid components. Remarkably, the ion signal of FA 18:1 (detected *m/z*: 281.2485; theoretical *m/z*: 281.2486) in the GPR3/G_s_ complex was significantly higher than that in the S1PR1/G_i_ complex and substantially higher than that in the buffer control (Fig. 2a, right panel; Supplementary information, Fig. S3h), suggesting its involvement in the complex. Besides mass measurements, the location of carbon–carbon double bond (C=C) in FA 18:1 was determined via the Paternò-Büchi (PB) derivatization and tandem MS (PB-MS/MS) (Fig. 2d). OA, having the C=C at the n-9 location, was identified as the dominant component (92%) (Fig. 2c). These findings strongly suggest that the lipid signal detected in the GPR3/G_s_ complex corresponds to OA, confirming the specificity of the OA signal in the GPR3 complex.

### Ligand-binding pocket of GPR3

Based on the above MS analysis, we modeled OA into the cryo-EM electron density in the pocket. OA fits the density well (Fig. 3a, b). The ligand-binding pocket is formed by the extracellular side of TMs 2, 3, 5, 6 and 7. A hydrophobicity analysis showed that the ligand-binding pocket is highly hydrophobic, matching well with the hydrophobic acyl chain of OA (Supplementary information, Fig. S3c). The hydrophobic residues L93^2.57^, V187^ECL2^, L283^7.39^, L116^3.32^, F263^6.51^, F120^3.36^, W260^6.48^, A117^3.33^, L195^5.40^, L198^5.43^, I124^3.40^ and F202^5.47^ (superscripts refer to the Ballesteros-Weinstein numbering^31^) (Fig. 3b) form a hydrophobic tunnel which penetrates the receptor from the extracellular side to the middle of lipid bilayer on the TM4, TM5 side at an angle of 45°. The tunnel is clearly visible from the top of the receptor (Supplementary information, Fig. S3d, e). Specifically, hydrophobic residues L116^3.32^, A117^3.33^, F120^3.36^, L198^5.43^, T121^3.37^ and A201^5.46^ make close contacts with the acyl chain of OA (Fig. 3c). The top of the pocket exhibits slight hydrophilicity, with two polar residues, Y280^7.36^ and H96^2.60^, situated at the extracellular entry of the pocket, facing the carboxyl group of OA (Fig. 3b). We also observed densities of two water molecules on the entry of the pocket, which connect Y280^7.36^ and H96^2.60^ to the carboxyl group of OA. When aligning the sequences of essential residues within the ligand-binding pocket, it becomes evident that this pocket is conserved across GPR3, GPR6, and GPR12 (Supplementary information, Fig. S6d).

To validate our structural observations, we conducted mutagenesis studies. Initially, we tested the ligand-binding pocket mutants without adding any exogenous ligand, as OA is naturally abundant in the cell culture media. Remarkably, mutations of Y280A^7.36^ and H96A^2.60^ both resulted in a significant decrease in receptor activity in the cAMP assay (Fig. 3d). Moreover, double mutations of Y280A^7.36^/H96A^2.60^ completely abolished the receptor activity in the cAMP assay, underscoring the crucial anchoring role of these polar residues in locking the ligand within the pocket. Likewise, we examined mutants of the hydrophobic tunnel, namely F120A^3.36^, W260A^6.48^, L283F^7.39^, and A201W^5.46^. All of these mutations substantially decreased receptor activity in the cAMP assay (Fig. 3d). In addition, mutants of other hydrophobic residues, such as V113F, L93F, I124F and V187F, also exhibited a decrease of receptor activity (Supplementary information, Fig. S6a). Notably, the W260A^6.48^ mutation caused a total abolition of receptor activity, highlighting the critical role of the Toggle Switch in receptor activation. Interestingly, residue A201^5.46^ is located at the end of the tunnel, directly connecting to the membrane lipid bilayer. The A201W^5.46^ mutation severely impaired receptor activation, suggesting that a lipid ligand may utilize A201 as a lateral entry point to bind the receptor (Fig. 3b). Of note, all mutants were expressed at a similar level to the WT receptor (Supplementary information, Fig. S4a).

To assess the responses of these mutants to exogenously added OA, we introduced OA at different doses into the medium of cells transiently transfected with the mutants. However, due to the high level of endogenous FAs, including OA, and the hypersensitivity of transiently transfected receptors, we encountered difficulties in obtaining reproducible dose-response data for these mutants with OA. To address this issue, we established AD293 cell lines that stably expressed each mutant. To ensure impartiality, we screened 24 colonies for each mutant and selected the colony with the highest expression level and maximal response to OA as the representative colony for that mutant (see Materials and Methods for details). The data obtained from these stable cell lines revealed that although the variation in EC_50_ values was small, all mutants F120A^3.36^, W260A^6.48^, L283F^7.39^, A201W^5.46^, Y280A^7.36^, H96A^2.60^ and H96A^2.60^/Y280A^7.36^ exhibited a lower E_max_ than the WT receptor. Particularly, F120A^3.36^ and W260A^6.48^ mutants completely lost their response to OA (Fig. 3e; Supplementary information, Table S3), which is consistent with the data obtained from transiently transfected mutants for endogenous ligand response. The majority of stably expressed GPR3 mutants showed similar expression levels, while W260A^6.48^ and H96A^2.60^/Y280A^7.36^ mutants exhibited relatively lower expression levels (Supplementary information, Fig. S4c). In a titration experiment, we demonstrated that the WT receptor expressed at the same level as the W260A^6.48^ and H96A^2.60^/Y280A^7.36^ mutants did not exhibit the altered activity (Supplementary information, Fig. S4b), indicating that the lack of receptor activity in these mutants is intrinsic to their nature and not influenced by the expression levels.

To further validate the position of OA in the ligand-binding pocket, we employed molecular dynamics (MD) simulations. We conducted triplicate runs with a duration of 200 ns each. Remarkably, within just a few picoseconds after the start of the simulation, OA rapidly stabilized within the ligand-binding pocket. Although the head and tail of OA exhibited fluctuations during the simulation, the overall position of OA remained similar to its initial pose in the cryo-EM structure (Supplementary information, Fig. S3f and Video S1). In addition to MD simulations, we utilized molecular docking to investigate the position of OA in the ligand-binding pocket. The results revealed that the top 5 scored poses from the docking simulations closely resembled the initial pose of OA in the cryo-EM structure (Supplementary information, Fig. S3g). This finding once again suggests that the position of OA within the ligand-binding pocket is energetically favorable.

### Ligand specificity of GPR3

OA is an abundant free fatty acid (FFA) in cells, and other common FFAs include stearic acid (STE), PAL and lauric acid (LAU). They all have a similar single acyl chain and differ from each other only by length of acyl chain or number of double bond;^32^ for instance, there is only one double bond difference between OA (FA 18:1, n-9) and STE (FA 18:0) (Supplementary information, Fig. S5a). In addition, lysophospholipids are also one acyl chain lipids but with a highly negatively charged head, and many lysophospholipids serve as signaling molecules through binding of GPCRs.^33^ Some of lysophospholipids, such as S1P and lysophosphatidic acid (LPA), have been proposed as ligands of GPR3.^1^ We therefore assessed the ligand specificity of GPR3.

**Fig. 4:**
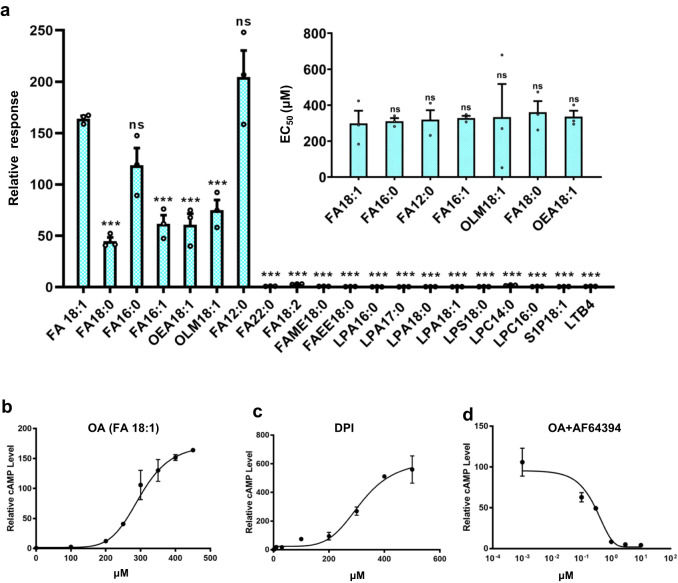
Ligand specificity of GPR3. **a** Comparison of GPR3 activation by lipids in a GPR3 stably expressing AD293 cell line via the GloSensor cAMP assay. The main panel, maximum response of various lipids. The right upper panel, EC_50_ of lipid ligands that activate GPR3. Data are presented as means ± SEM; *n* = 3 independent samples. Maximum response for FA 12:0, 16:1, 18:0, OEA 18:1, OLM 18:1 at 500 μM; FA 18:1 at 450 μM; FA 16:0 at 400 μM; FA 22:0, 18:2 at 300 μM, FAME 18:0, FAEE 18:0 and all lysophospholipids at 100 μM; LTB4 at 3 μM. Data are presented as means ± SEM; *n* = 3 independent samples; n.s. not significant; *P < 0.05; **P < 0.01; ***P < 0.001. Two-sided *t*-test. **b**–**d** Dose-response curves of OA (**b**), DPI (**c**) and AF64394 (**d**) in a GPR3 stably expressing AD293 cell line by the GloSensor cAMP assay. Data are presented as means ± SEM; *n* = 3 independent samples. OA concentration was fixed at 300 μM. The *R* values for curve fit are 0.9778 (**b**), 0.937 (**c**), and 0.9077 (**d**), respectively.

In the past, one major challenge in identifying the ligand for GPR3 is inconstancy of functional assays which generated a mixed signal of GPR3 activation.^1,3,6^ The lack of reproducibility in these assays might be attributed to the interference from endogenous lipids or the variations in GPR3 expression level. To overcome this obstacle, as mentioned above, we developed an AD293 cell line that stably expresses GPR3 (Supplementary information, Fig. S5d) along with a GloSensor of cAMP (details see Materials and Methods). This AD293 cell line exhibited a robust response to OA stimulation (Fig. 4b), which demonstrated excellent reproducibility, allowing us to conduct a systematic analysis of GPR3’s ligand specificity. Upon stimulation with OA (FA 18:1), the GPR3 stably expressing cell line displayed a typical dose-dependent induction of cAMP, which reached the highest level at a concentration of 450 μM (Fig. 4a, b for activity; Supplementary information, Fig. S5a for chemical structure). STE (FA 18:0), only one double bond difference from OA, only had about 1/3 of the E_max_ of OA. PAL (FA 16:0), two carbons shorter than OA, had a slightly lower E_max_ than OA. Intriguingly, palmitoleic acid (PML, FA 16:1), with one additional double bond compared to PAL, had only half of the E_max_ of PAL. Oleamide (OLM, 18:1), differing from OA only by an amine group at the head, displayed only half of the E_max_ of OA. Similarly, oleoylethanolamide (OEA, 18:1), which has one ethanolamine modification at the head of OA, exhibited only half of the E_max_ of OA. Surprisingly, LAU (FA 12:0), which contains only 12 carbons in the acyl chain, showed a higher E_max_ than OA. In contrast, behenic acid (BEH, FA 22:0), which has a longer acyl chain tail, showed no activity at all. Linoleic acid (LIN, FA 18:2), differing from OA by only one additional double bond, exhibited no activity in the cAMP assay. Furthermore, we observed that a methyl or ethyl modification of STE (methyl stearate (MES), FAME 18:0; ethyl stearate (ETS), FAEE 18:0) completely abolished receptor activation in the cAMP assay (Fig. 4a for activity; Supplementary information, Fig. S5a for chemical structure).

Since the ligand-binding pockets of GPR3, GPR6 and GPR12 are conserved, we tested whether GPR6 and GPR12 have similar responses to FFAs. For this purpose, we established AD293 cell lines that stably express GPR6 or GPR12. The cAMP assay showed that GPR6 and GPR12 could also be activated by a subset of FFAs but with different fingerprints; namely, PML (FA 16:1) and LAU (FA 12:0) strongly activated GPR6, while OA showed a strongest effect on GPR12 activation (Supplementary information, Fig. S6e, f).

Despite having similar acyl tails, none of the lysophospholipids that we tested exhibited cAMP induction activity in our GPR3 stably expressing AD293 cell line. This includes LPA (16:0, 17:0, 18:0, 18:1), LPS (18:0), LPC (14:0, 16:0), and S1P (Fig. 4a for activity; Supplementary information, Fig. S5a for chemical structure). Additionally, leukotriene B4 (LTB4) also failed to activate GPR3 in the cAMP assay. We next compared the dose-response and EC_50_ of lipids that did activate GPR3 (Fig. 4b; Supplementary information, Fig. S5b and Table S2). The data showed that OA had a similar EC_50_ of 300 μM to other tested lipids, which is in the middle range of the reported human plasma OA concentration (0.03–3.2 mM).^34^ We also examined a synthesized GPR3 agonist, DPI.^4^ In spite of a higher E_max_, DPI showed a similar EC_50_ (314.8 μM) to OA (Fig. 4c). Conversely, AF64394, a synthetic antagonist of GPR3,^35^ strongly inhibited OA-induced receptor activation with an IC_50_ of 0.171 μM (Fig. 4d). Molecular docking revealed that both DPI and AF6394 can effectively fit within the ligand-binding pocket of GPR3 (Supplementary information, Fig. S6b, c).

### Structural basis of ligand specificity

**Fig. 5:**
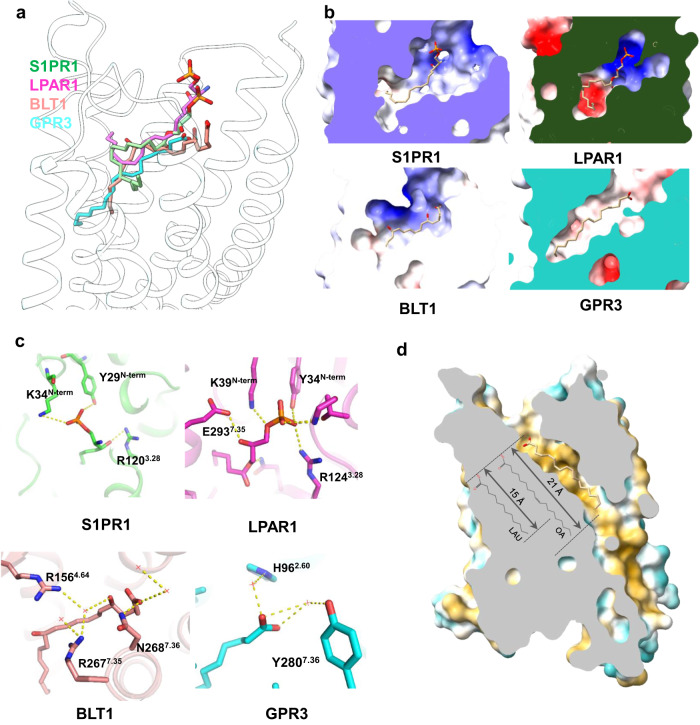
Comparison of receptor binding mode of OA with those of LPA, S1P and LTB4. **a** Superimposition of OA with LPA (PDB: 7td0), S1P (PDB: 7wf7) and LTB4 (PDB: 7vkt) in GPR3. **b** Electrostatic analysis of ligand-binding pocket of S1PR1, LPAR1, BLT1 and GPR3. Blue color indicates positive charge and red color indicates negative charge. **c** The detailed polar interactions between lipid head group and receptor. **d** Measurement of the hydrophobic tunnel of GPR3. The receptor was drawn in hydrophobic surface mode in Chimera. The yellow color indicates hydrophobicity and the blue color indicates hydrophilicity.

We next investigated the structural basis of ligand specificity of GPR3. Despite having similar long acyl tails to FFAs, lysophospholipids failed to induce cAMP activation in the GPR3 stably expressing AD293 cell line. This suggests that the head of lysophospholipids engage the receptor differently. A comparison of OA-bound GPR3 with S1P-bound S1PR1,^36^ LPA-bound LPAR1^37^ and LTB4-bound Leukotriene B4 receptor 1^38^ (BLT1) showed that while the hydrophobic tails occupy similar positions of the receptor, the binding modes of the polar head group are largely different (Fig. 5a). The phosphate head groups of both S1P and LPA carry a strong negative charge, fitting well with the positively charged upper region of the ligand-binding pocket of their receptors (Fig. 5b). The head of LTB4 is less negatively charged (absence of phosphate group); however, two hydroxyl groups at C5 and C12 positions make the middle ridge of LTB4 slightly negatively charged, which matches with the slightly positively charged upper region of the ligand-binding pocket of BLT1. In contrast, the ligand-binding pocket of GPR3 is almost completely neutral (Fig. 5b). The difference in electrostatic distribution between lysophospholipid receptors and GPR3 indicates that charged lipids (e.g., S1P, LPA, and LTB4) are not compatible with the ligand-binding pocket of GPR3.

Upon closer examination of the interactions between ligands and receptors, we noted that in S1PR1 and LPAR1, the phosphate head group of the ligand engages in polar interactions with crucial tyrosine and lysine residues located in the N-terminal cap alongside an arginine at position 3.28.^36,37^ In addition, several other polar residues within the ligand-binding pocket also participate in the interactions (Fig. 5c). In BLT1, a hydrogen-bond network connects key polar residues, water molecules and ligand together^38^ (Fig. 5c). In contrast, there is no direct polar interaction between the carboxyl group of OA and GPR3, and only two water molecules connect the carboxyl head of OA to H96 and Y280 (Fig. 5c). Collectively, the above analyses suggest that a charged pocket and a network of polar interactions are essential for the engagement of highly charged lipids, such as S1P, LPA and LTB4, to their receptors. Conversely, highly charged lipids are not GPR3 ligands due to the complete hydrophobicity of the ligand-binding pocket of GPR3.

We then investigated the structural basis of FFA selectivity. OA’s 18-carbon acyl chain fits snugly in the ~21 Å long hydrophobic tunnel in the ligand-binding pocket (Fig. 5d). The minimal-length acyl chain we tested that can fully activate GPR3 is LAU with 12 carbons. The 15 Å long LAU acyl chain fits well within the hydrophobic tunnel (Fig. 5d), and docking simulations of LAU into the ligand-binding pocket of GPR3 suggest that its acyl tail utilizes most hydrophobic residues in the tunnel to activate the receptor, including W260^6.48^, the sensor of lipid ligand binding (Supplementary information, Fig. S5c). Calculating from the length of the hydrophobic channel (~21.8 Å) (Supplementary information, Fig. S3d), the maximal-length FA that can fit in the tunnel is 18-carbon acyl chain (~21 Å) (Fig. 5d). In support of this, the 22-carbon BEH (FA 22:0) failed to activate the receptor (Fig. 4a). Similarly, ETS (FAEE 18:0) and MES (FAME 18:0), which have only an ethyl or a methyl group added to the carboxyl head of STE, respectively (Supplementary information, Fig. S5a), also failed to activate the receptor (Fig. 4a). Surprisingly, LIN (FA 18:2, n-9,12), with only one additional double bond at C12 position compared to OA (FA 18:1, n-9), also failed to activate the receptor, suggesting that the hydrophobic tunnel in the pocket cannot accommodate the bending of the acyl chain caused by two double bonds. Intriguingly, PML (FA 16:1, n-9), which differs from PAL (FA 16:0) by only one double bond at position C9, substantially decreased receptor activation compared to PAL. This may be due to the greater restrictive effect of the double bond on the shorter 16-carbon acyl chain of PML than on the longer 18-carbon acyl chain of OA.

### The activation of GPR3 by OA via cold stimulation

**Fig. 6:**
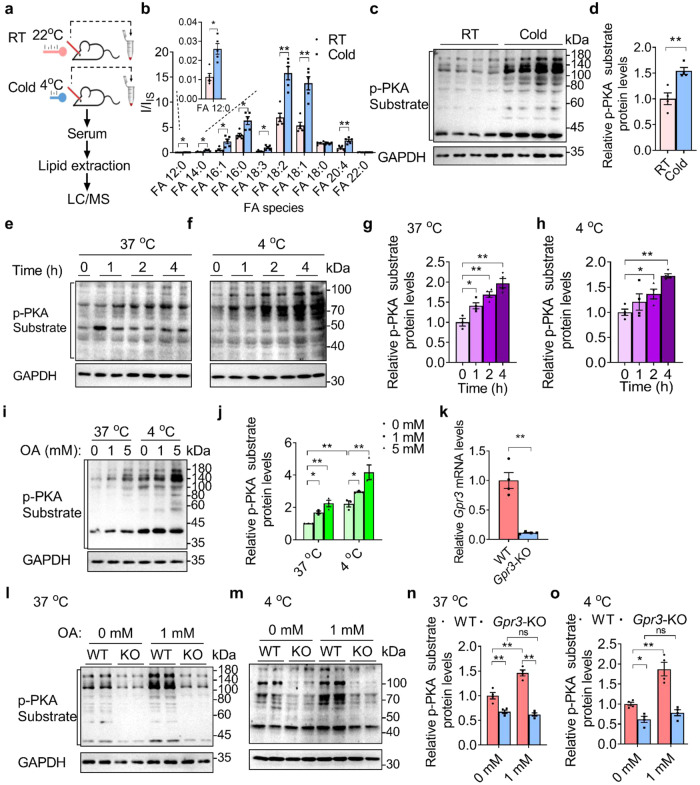
OA activates the G_s_/cAMP/PKA signaling through GPR3 in brown adipose tissue. **a** Scheme of RT/cold treatment and LC-MS analysis procedure. **b** LC-MS analysis of serum FAs in mice with acute cold stimulation (6 h) (*n* = 5 per group). **c**, **d** p-PKA substrate and GAPDH levels in iBAT of mice with acute cold stimulation (6 h) were measured by immunoblotting and quantified by densitometry using ImageJ (*n* = 4 per group). **e**–**h** iBAT was isolated from C57BL/6 mice at 4–6 weeks old and cut into 10 mg pieces. 10 mg iBAT was further cut into 4 pieces. iBAT pieces were incubated at 37 °C or 4 °C in DMEM medium, and treated with OA (1 mM) for different times (0 h, 1 h, 2 h and 4 h). p-PKA substrate and GAPDH levels were measured by immunoblotting (**e**, **f**), and quantified by densitometry using ImageJ (**g**, **h**) (*n* = 3–4 per group). **i**, **j** Another group of iBAT pieces were incubated at 37 °C or 4 °C in DMEM medium, and treated with different doses of OA (0 mM, 1 mM, 5 mM) for 4 h. p-PKA substrate and GAPDH levels were measured by immunoblotting, and quantified by densitometry using ImageJ (*n* = 3 per group). **k** *Gpr3* mRNA levels were measured in the iBATs of WT and *Gpr3* KO mice at 7 weeks old (*n* = 4 per group). **l**–**o** iBATs were isolated from WT and *Gpr3* KO mice at 4–6 weeks old and cut into 10 mg pieces. 10 mg iBAT was further cut into four pieces. iBAT pieces were incubated at 37 °C or 4 °C in DMEM medium, and treated with or without OA (1 mM) for 4 h. p-PKA substrate and GAPDH levels were measured by immunoblotting, and quantified by densitometry using ImageJ (*n* = 4 per group). Data are presented as means ± SEM. *n* = 3–5 independent samples. Student’s *t*-test was used to compare the differences between two groups. The differences between three groups were examined using one-way ANOVA and LSD *t*-test. *P < 0.05. **P < 0.01.

The previous adipocyte thermogenic study suggested that cold stimulates lipolytic signals to induce GPR3 expression through a transcriptional program.^16^ We asked whether cold stimulates the secretion of lipid molecules, particularly certain FFAs, to directly activate GPR3. For this purpose, we divided mice into two groups, one group kept at room temperature (RT) and the other group exposed to cold at 4 °C for 6 h. Then we collected blood samples from both groups and extracted lipids from the serum, and then performed an LC-MS analysis of the lipid component (Fig. 6a). The results showed that the cold stimulation significantly increased the levels of OA (FA 18:1), LIN (FA 18:2), LAU (FA 12:0), PAL (FA 16:0) and PML (FA 16:1), compared to the samples from mice kept at RT (Fig. 6b). Most interestingly, OA and LAU exhibited the highest E_max_ in our GPR3 activation study (Fig. 4a). Of note, the level of LAU is two magnitudes less than that of OA, which explains why only OA was detected in the initial GPR3/G_s_ complex in the MS analysis. In contrast, STE (FA 18:0) from cold-stimulated mouse serum did not show an increase over RT samples. These findings indicate that cold stimulation strongly induces the secretion of OA, which is capable of activating GPR3.

In conjunction with the induction of OA after cold stimulation, the G_s_/cAMP/PKA signaling pathway (as measured by the total phosphorylation of PKA substrates using western blot) in iBAT from mice exposed to cold at 4 °C for 6 h was markedly activated (Fig. 6c, d). We next asked whether cold-induced OA can activate G_s_/cAMP/PKA pathway in a physiological setting. We added OA to iBAT isolated from 6-week-old mice at 4 °C and examined the overall phosphorylation of PKA substrates, a hallmark of G_s_/cAMP/PKA signaling. Remarkably, we observed a robust increase of G_s_/cAMP/PKA signaling in response to OA stimulation in a time- and dose-dependent manner (Fig. 6f, h–j). In contrast, we also observed an increase of G_s_/cAMP/PKA signaling in iBAT of mice treated at 37 °C, but to a lesser extent than in the samples treated at 4 °C (Fig. 6e, g, i, j). To test the specificity of OA, we added STE (FA 18:0) and LIN (FA 18:2) to the iBAT of WT mice at 4 °C. However, neither STE nor LIN induced G_s_/cAMP/PKA signaling (Supplementary information, Fig. S7a), while OA strongly induced phosphorylation of PKA substrate, consistent with our previous observation that STE had a weak activity and LIN had almost no activity on GPR3 (Fig. 4a).

We then asked whether the enhancement of G_s_/cAMP/PKA signaling via OA at the cold condition is mediated by GPR3. For this purpose, we generated *Gpr3* KO mice (Fig. 6k) (see Materials and Methods for details) and compared the G_s_/cAMP/PKA signaling in WT iBAT with that in *Gpr3* KO iBAT. As expected, OA substantially increased G_s_/cAMP/PKA signaling in WT iBAT, whereas the induction of G_s_/cAMP/PKA signaling by OA was completely blocked in *Gpr3* KO iBAT (Fig. 6l–o). We also tested whether cold induces an increase of OA level in *Gpr3* KO mice, and the LC-MS analysis showed that the KO of *Gpr3* gene in mice does not affect OA secretion under cold stimulation (Supplementary information, Fig. S7b). Collectively, these data demonstrated that OA activates the G_s_/cAMP/PKA signaling through GPR3 in iBAT under cold stimulation.

### A “born to be activated and cold to enhance” model for GPR3 activation

**Fig. 7:**
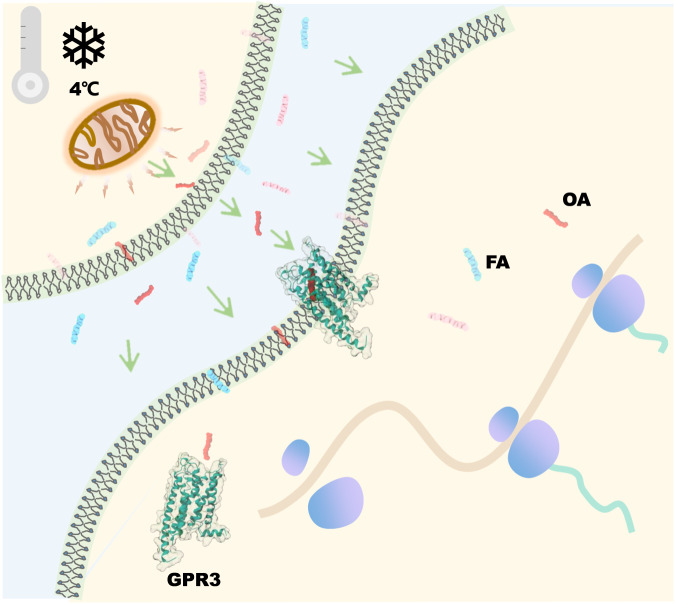
A “born to be activated and cold to enhance” model for GPR3 activation. The worm-like shapes represent FAs existing in cytoplasma, membrane or extracellular medium of cells.

The autonomous activation of GPR3 has long been a perplexing enigma in the field. The structural information of OA-bound GPR3 enables us to solve the riddle. The ligand-binding pocket of GPR3 is widely open at the extracellular side (Fig. 1a), and it is connected to a hydrophobic tunnel that extends from the extracellular region to the middle of the lipid membrane at the TM4, TM5 side (Supplementary information, Fig. S3c–e). Interestingly, AlphaFold2^39^ prediction of apo GPR3 also reveals the presence of a similar tunnel (Supplementary information, Fig. S8a, b), suggesting that the tunnel is an inherent characteristic of GPR3. The presence of this two-ended open hydrophobic tunnel allows FFAs, like OA, to bind and interact with the receptor either from the extracellular side (orthodox entry) or the membrane side (lateral entry), as FFAs are major constituents of the lipid bilayer of the membrane.^40,41^ Considering that OA, along with other FFAs, are abundant in cells, we propose that GPR3 is a “born to be activated” receptor. Shortly after translation from mRNA by ribosomes, GPR3 can readily bind OA from either the cytoplasm or the membrane, thus becoming activated at the plasma membrane (Fig. 7). Moreover, since FFAs are also abundant in the extracellular medium of cells, such as in the bloodstream, GPR3 can be activated after being transferred to the plasma membrane (Fig. 7). Furthermore, in cells involved in energy expenditure and metabolic process, for instance, the thermogenic adipocytes, cold stimulates the lipolysis process to generate a large amount of OA (up to 3.2 mM), LAU et cetera, which in turn are secreted to the extracellular side of cells.^34,42–44^ In this scenario, OA, as a major component of the secreted lipolytic signals, binds to and activates the membrane-embedded GPR3 (Fig. 7). Based on this, we propose a “born to be activated and cold to enhance” model for GPR3 activation. In this model, GPR3 is intrinsically predisposed to bind and be activated by the abundant FFAs within cells, and this activation is further amplified by the cold-stimulated secretion of OA.

## Discussion

In this study, we identified OA as an endogenous ligand of GPR3. Although the observed EC_50_ of 300 μM for OA may seem relatively high, it is noteworthy that the normal range of plasma OA concentration in humans varies from 0.03 mM to 3.2 mM.^34^ Moreover, the typical plasma FFA concentration is 0.2–1.5 mM. The concentration of FFAs in blood can vary depending on various factors such as the individual’s metabolic state, diet, and physical activity level. During times of increased lipolysis (the breakdown of fat for energy), such as exercise, cold stimulation or fasting, the concentration of FFAs in blood can increase substantially.^42,43^ In some pathological conditions such as diabetes or obesity, the concentration of FFAs in blood may also be elevated.^42,44^ Considering that the EC_50_ of 300 μM for OA falls within the middle range of plasma OA concentration (0.03–3.2 mM), we elucidated how GPR3 can sensitively respond to increased OA signals during cold stimulation (Fig. 6b) and effectively trigger the G_s_/cAMP/PKA signaling to initiate thermogenesis (Fig. 6c). Furthermore, recent research on GPR120 revealed that the EC_50_ values of OA and LIN for GPR120 are 40.6 μM and 57.4 μM, respectively,^45^ indicating that certain receptors can indeed be activated by FAs at submillimolar concentrations.

The highly constitutive G_s_ coupling activity of GPR3 in thermogenic adipocytes is a desirable trait for energy expenditure and metabolic regulation, especially in the context of obesity and other metabolic disorders. Although the precise mechanisms underlying how lipolytic signals induce GPR3 expression remain unclear, our data demonstrate that cold stimulation in mice leads to a significant release of OA into the bloodstream (serum). Then, OA acts as a ligand to activate GPR3, resulting in robust G_s_/cAMP/PKA signaling that drives thermogenesis. In contrast to saturated STE, OA has been known to exert beneficial effects on health for quite some time. For instance, feeding rats with OA induces thermogenesis and reduces body weight,^46,47^ though the underlying mechanism was previously unknown. Our data have now directly connected OA to GPR3 activation and thermogenesis, providing insights into this long-standing puzzle. Furthermore, previous GPR3 thermogenesis study suggesting that the highly abundant endogenous ligand in cells could be the ligand of GPR3^16^ is compatible with our finding.

Taken together, we identify OA as an endogenous ligand of GPR3, shed light on the ligand recognition mechanism of GPR3, provide compelling evidence that cold stimulates the secretion of OA as the ligand to activate GPR3 in mice and propose a “born to be activated and cold to enhance” model for GPR3 activation. These structural and biological information revealed by our study serves as a starting point for the understanding of the constitutive signaling of GPR3 in cold-driven thermogenesis.

## Materials and methods

### Constructs

Full-length GPR3 was code-optimized and subcloned into pFastBac and fused with the large part of NanoBiT (LgBiT),^48^ followed by a tobacco etch virus (TEV) cleavage site and 2× maltose-binding protein (MBP) fusion tag. The C-terminus of human Gβ_1_ was fused with a renovated high-affinity small part of NanoBiT (HiBiT). A human Gα_s_ with two dominant negative mutations (G226A and A366S) and a human Gγ_2_, were separately subcloned into pFastBac.

### Expression and purification of the complex

We used a similar expression and purification process as described before.^24^ Briefly, baculovirus encoding GPR3–LgBiT–TEV–2× MBP, Gα_s_, Gβ_1_, and Gγ_2_ proteins were co-infected into Sf9 cells. Two days later, cells were collected and resuspended in a lysis buffer containing 20 mM HEPES, pH 7.5, 150 mM NaCl, 10 mM MgCl_2_, 20 mM KCl and 5 mM CaCl_2_. The mixture was incubated for 90 min at RT with apyrase (0.5 mU/mL) and Nb35 (20 µg/mL). Then, membrane was solubilized in buffer of 0.5% (w/v) lauryl maltose neopentylglycol (LMNG; Anatrace) and 0.1% (w/v) CHS-Tris for 2 h at 4 °C, followed by ultracentrifugation at 45,000 rpm at 4 °C for 45 min twice. The supernatant was loaded on an amylose column for 2 h, flowed out, washed with buffer solution containing 25 mM HEPES, pH 7.5, 150 mM NaCl and 0.01% LMNG/0.002% CHS, and eluted by the same wash buffer added with 10 mM maltose. After concentration and TEV cleavage overnight at 4 °C, the complex protein was loaded onto a Superdex 200 Increase 10/300 GL (GE Health Sciences) gel infiltration column pre-equilibrated with the buffer of 25 mM HEPES, pH 7.5, 150 mM NaCl, 0.00075% (w/v) LMNG, 0.00025% glyco-diosgenin and 0.0002% CHS (w/v) (Anatrace). The complex fractions were concentrated to 10 mg/mL and snap-frozen for grid preparation.

### Expression and purification of Nb35

As described before,^25,49^ briefly, BL21 cells expressing Nb35 were cultured in TB media with 0.1% glucose, 1 mM MgCl_2_ and 50 μg/mL ampicillin at 37 °C until OD600 reached 0.7. Then cells were induced by 1 mM IPTG, and cultured at 28 °C for 4 h. The cells were harvested by centrifugation at 39,000 rpm for 20 min, and the precipitate was lysed in TES buffer containing 0.2 M Tris, pH 8.0, 0.5 mM EDTA and 0.5 M sucrose by stirring at 4 °C for 3 h, followed by ultracentrifugation at 45,000 rpm at 4 °C for 45 min twice. The supernatant was then incubated with nickel column for 2 h at 4 °C, and washed with 20 column volumes of washing buffer (20 mM HEPES, pH 7.5 and 150 mM NaCl) and again with high salt buffer (43 mM NaH_2_PO_4_·H_2_O, 7 mM NaH_2_PO_4_ and 1 M NaCl, pH 6.0). Nb35 protein was eluted by 10 column volumes of buffer containing 19 mM NaH_2_PO_4_·H_2_O, 38 mM NaH_2_PO_4_, 150 mM NaCl and 250 mM imidazole, pH 7.0. After being concentrated, the protein was then loaded onto a Superdex 200 Increase 10/300 GL (GE Health Sciences) gel filtration column pre-equilibrated with the buffer of 20 mM HEPES, pH 7.5, and 150 mM NaCl. The purified protein was concentrated to 10 mg/mL, supplemented with 10% glycerol and stored at −80 °C until use.

### Grid preparation and cryo-EM data collection

A 3–5 µL protein complex sample (~10 mg/mL) was applied to a glow-charged quantifoil R1.2/1.3 Cu holey carbon grids (Quantifoil GmbH), followed by vitrification in liquid ethane on a Vitrobot Mark IV (Thermo Fisher Scientific) instrument at setting of blot force 10, blot time 5 s, humidity 100%, temperature 4 °C. Grids with evenly distributed particles in thin ice were loaded to a FEI 300 kV Titan Krios TEM with a Gatan Quantum energy filter. Images were captured by a Gatan K2 Summit direct electron detector with a super-resolution counting model at pixel size of 0.55 Å (magnitude of 64,000×). The energy filter slit was set to 20 eV. Each image contains 40 frames with a total exposure time of 7.3 s at a dose rate of 1.5 e/Å^2^/s (total dose of 60 e/Å^2^), nominal defocus value varying from −1.2 µm to −2.2 µm.

### Data processing

We use a similar pipeline for data processing as described before.^24,36^ Briefly, 2397 raw movies were binned once (1.1 Å) and motion-corrected by MotionCor2,^50^ followed by CTF estimation via CTFFIND 4.1.^51^ About 1.3 × 10^6^ particles were picked by crYOLO,^52^ followed by reference-free 2D classification in RELION.^53^ About 644,000 particles of well-defined 2D features were used for initial model generation (cryoSPARC^54^ ab initio) and 3D classification. The model was used as reference in RELION 3D classification (~5 classes). The best class (~386,000) with clear secondary structure features was selected for a 3D refinement in RELION, followed by a Baysian polishing,^55^ a 3D refinement and a CTF refinement in RELION. Then it was subjected to a second round 3D classification (3 classes) with mask on the complex to yield a class of ~288,000 particles for final refinement by the cryoSPARC Non-uniform Refinement, which generated a map of 2.79 Å, based on the gold standard FSC = 0.143 criterion. Local resolution estimations were performed using an implemented program in cryoSPARC.

### Model building

AlphaFold^39^ prediction of human GPR3 (AF-P46089-F1) and G_s_ protein complex from M1R (PDB: 7f4d)^49^ were used as initial models for model rebuilding against the electron microscopy map. Models were docked into the electron microscopy density map by UCSF Chimera^56^ and then subjected to iterative manual adjustment in Coot,^57^ followed by a rosetta cryoEM refinement^58^ and Phenix real space refinement.^59^ Structural figures were prepared in UCSF Chimera, ChimeraX^60^ and PyMOL (https://pymol.org/2/). Ligand–receptor interaction was plotted by the LigPlot.^61^

### MD simulation

The close to the end cryo-EM structure of GPR3 (receptor only) was used as initial model in the MD simulation. The ICL3 break (232–240) was filled with 5 alanine residues. The model was prepared and parameterized in CHARMM-GUI.^62^ Protonation states of all titratable residues were assigned at pH 7.0. Histidine was modeled as neutral. The GPR3 model was inserted into a bilayer lipid containing POPC (palmitoyl-2-oleoyl-sn-glycero-3-phosphocholine) and cholesterol at ratio of 4:1, the membrane size is 65 Å × 65 Å with 22.5 Å water in the top and bottom (final system dimensions: ~65 Å × 65 Å × 120 Å). Ion was set to 0.15 M KCl. The Amber force fields were set to: protein FF19SB, lipid LIPID17, water TIP3P and ligand GAFF2. The simulations were performed by Amber20 package.^63^ The system was first energy minimized for solvent and all atoms, heated to 300 K in 300 ps and then equilibrated for 700 ps, followed by three independent production runs of 200 ns with a time step of 2 fs. During simulations, Particle mesh Ewald algorithm was applied for the calculation of long-range electrostatic interaction and a cutoff of 10 Å was applied for short-range electrostatic interaction and van der Waals interactions. All bonds with hydrogens were constrained by SHAKE algorithm. The system temperature (300 K) and pressure (1 atm) were controlled by Langevin thermostat and Berendsen barostat, respectively. The trajectories were analyzed and visualized in VMD^64^ and video was recorded by VMD.

### Molecular docking

The docking method is similar to the previous study.^38^ Briefly, the cryo-EM structure of GPR3 (receptor only) including two water molecules was used as the template. The model was prepared and minimized as before. OA was placed in the ligand-binding pocket using the triangle matcher with London docking score. Refinement was employed based on a rigid receptor and GBVI/WSA docking scoring. The top-scored poses and statistics were shown in Supplementary information, Fig. S3g.

### MS analysis

The sample solution (100 µL, ~1.5 mg/mL protein complex or buffer) was added with [D4] FA 18:0 (2 µL, 1 mM) as the internal standard and was placed in a 2-mL vial. It was later mixed with a solution of 1365 µL ice-cold methyl tert-butyl ether/methanol/2 M hydrochloric acid solution (200:60:13, v/v/v) and vortexed for 1 min. Then, 250 µL of 0.1 M HCl was added and vortexed for 5 min. After centrifugation (10,000 rpm, 5 min), the upper phase of solution was obtained, dried under nitrogen flow, and redissolved in 100 µL methanol/1 M NH_3_·H_2_O solution (9:1, v/v). The solution was then subjected to LC-MS analysis in negative ion mode with three technical repeats and three biological repeats. The lipid extracts were subjected to the PB derivatization to determine the location of C=C. Detail of the derivatization has been reported. In brief, the dried lipid extract and 10 mM 2-acetylpyridine were dissolved in 150 µL acetonitrile. The solution was irradiated by 254 nm UV for 15 s in a flow microreactor and ~50 µL solution was collected for LC-MS analysis.^65^

The LC-MS system contained an Acquity l-class system (Waters, Milford, MA, USA) hyphenated with a tims-time-of-flight of mass spectrometer (Bruker Daltonics, Bremen, Germany). A C18 column (150 mm × 2.1 mm, 1.6 µm) was used for lipid separation at a flow rate of 0.2 mL/min and the oven temperature was set at 55 °C. A binary buffer system was used with buffer A containing 20 mM ammonium acetate aqueous solution in acetonitrile/water (60/40, v/v) and buffer B containing isopropanol/acetonitrile (90/10, v/v). The mobile phase gradient was set as follows: 30%–70% B in 0–7 min, 70%–95% B in 7–10 min, 95% B in 10–12 min, 95%–30% B in 12–13 min, and 30% B in 13–15 min. The MS parameters were as follows: ESI voltage, –3000/+4500 V; dry temperature, 220 °C; dry gas, 8.0 L/min; collision RF, 400 Vpp, CID energy at 15 eV.

For examination of FA components in mouse serum, mouse plasma (15 µL and 30 µL of 10 µM [D4] FA 18:0 added as internal standard) was placed in a 2-mL vial, with 560 µL ice-cold water/methanol/2 M hydrochloric acid solution (250:300:10, v/v/v), and vortexed for 0.5 min. Then, 500 µL isooctane was added and vortexed for 5 min to extract FAs. After 5 min centrifugation, the upper solution was obtained. Isooctane extraction was repeated and upper solution from twice extraction was collected, dried under nitrogen flow, and redissolved in 60 µL methanol. The solution was then subjected to LC-MS analysis. The detection was under negative mode.

### GloSensor cAMP assay

The GloSensor cAMP assay was performed according to the instructions of Promega. AD293 cells were split into 6-well plate (300,000 cells/mL) and then transfected with the pGloSensor™-22F cAMP plasmid and pcDNA3-GPR3 plasmid by Lipofectamine 2000, at a ratio of 2:1 (μL:μg). 24 h after transfection, cells were split into 96-well plate for another 24 h incubation. After removal of cell medium, 90 μL of 3% v/v (the GloSensor cAMP reagent vs serum-free medium) substrate was added into each well. After 2 h incubation at RT, background measurements were taken, and then 10 μL 10× stock compounds were added for the second measurements of ligand activation via EnVision 2105 (PerkinElmer). Relative cAMP level was calculated by the cAMP level after addition of ligand divided by the cAMP level before addition of ligand (basal level). Stock FAs (i.e., 10–50 mM) were solubilized in DMSO, then diluted in cell culture buffer to designated concentrations and sonicated to ensure that they were evenly distributed in the buffer before being added to cells.

### Cell-based reporter assay

The CRE, SRE, SRF-RE, and NFAT-RE reporter assays (Promega) were performed according to the manufacturer’s instructions as described before.^19^ Briefly, AD293 cells were split into 24-well plate at a density of 40,000 per well and then transfected with 100 ng reporter, 10 ng pcDNA3-GPR3, 10 ng phRGtkRenilla plasmids (Promega) by X-tremeGENE HP (Roche) at a ratio of 3:1 over DNA amount after 1 day of growth on 37 °C at 5% CO_2._ 24 h after transfection, cells were harvested and lysed by addition of 1× Passive Lysis Buffer (Promega). The luciferase activity was assessed by the Dual-Glo Luciferase system (Promega). Data were plotted as firefly luciferase activity normalized to Renilla luciferase activity in Relative Luciferase Unit (RLU).

### Establishment of the stable cell line

AD293 cells were split into 6-well plate (300,000 cells/mL), and then transfected with the pGloSensor™-22F cAMP plasmid and the pcDNA6-GPR3-3× FLAG plasmid (WT receptor or the mutants, F120A^3.36^, W260A^6.48^, L283F^7.39^, A201W^5.46^, Y280A^7.36^, H96A^2.60^ and H96A^2.60^/Y280A^7.36^) by Lipofectamine 2000 at a ratio of 2:1 (μL:μg). Geneticin (2 mg/mL) and blasticidin (20 μg/mL) were used to select colonies that stably express both GPR3 and GloSensor 22F. The cell culture medium containing antibiotics was changed every 2 days till well-separated colonies appeared. Then individual single colony was picked by a small piece of sterilized filter paper soaked with trypsin and separately cultured in a single well of 24-well plate. At least 24 candidate colonies were selected for each mutant and WT. After several days of culture, each colony was triplicated. One of the triplicates was used for western blot analysis, another for the GloSensor cAMP assay, and the remaining one was stored for staining. The colonies with top scores in both expression (western blot) and functional assay were selected as the representative colonies for each mutant or WT for further analysis.

### Western blot

AD293 cells were transfected with 100 ng pcDNA3-GPR3-3× FLAG per well of 24-well plate by PEI at the ratio of 5:1 over DNA amount. Two days after transfection, cells were lysed by cell lysis reagent (Sigma), and proteins in cell lysates were separated in 10% Bis-Tris gels at 170 V for 1 h and then transferred onto polyvinylidene difluoride (PVDF) membranes at 100 V for 1.5 h. The membranes were blocked with 10% milk in TBS-T (20 mM Tris-HCl, pH 7.5, 50 mM NaCl, 0.1% Tween-20) at RT for 30 min. One of the membranes was incubated at RT for 2 h with monoclonal anti-FLAG M2-peroxidase (HRP) antibody (1:5000, Sigma) in TBS-T. The other one was incubated with β-actin mouse mAb (1:10,000, ABclonal) in TBS-T at RT for 2 h. After being washed with TBS-T, the membrane was incubated for 30 min with HRP goat anti-mouse IgG (1:5000, ABclonal) in TBS-T. After treatment with chemiluminescent substrate (Thermo Fisher Scientific), protein bands were detected by iBright CL1000 imaging system (Thermo Fisher Scientific).

### Animals

Male C57BL/6N mice were obtained from Charles River Laboratories (Beijing, China). Animal experiments were carried out in accordance with the Guide for the Care and Use of Laboratory Animals (8th edition) and approved by the Institutional Animal Care and Use Committee of Harbin Institute of Technology (HIT/IACUC).

### Cold stress experiments

Eight-week-old male C57BL/6N mice were purchased from Charles River (Beijing Vital River Laboratory Animal Technology Co., Ltd.). Mice were randomly divided into two groups. One group was kept at RT (22 °C). The other group was used for cold exposure experiments. Individual mouse was placed in a single cage in a cold room (4 °C) with free access to water for 6 h. Blood samples were collected from orbital sinus.^66^

### Ex vivo experiments

iBAT was isolated from C57BL/6N mice at 4–6 weeks old and cut into 10 mg pieces. 10 mg iBAT was further cut into four pieces.^67^ iBAT pieces were incubated in Dulbecco’s modified eagle medium (DMEM) at 4 °C, and treated with OA (1 mM) for different times (0 h, 1 h, 2 h, and 4 h). Another group of iBAT pieces were incubated in DMEM medium at 4 °C, and treated with different doses of OA (0 mM, 1 mM, 5 mM) for 4 h. OA (Sigma, O1008) was dissolved in serum-free DMEM (Gibco, high glucose, 12100046) by using an ultrasonic cleaner at 40 °C for 10 min. p-(S/T)PKA substrate and GAPDH levels were measured by immunoblotting, and quantified by densitometry using ImageJ. iBAT was also isolated from WT and *Gpr3* KO mice at 4–6 weeks old and cut into 10 mg pieces. 10 mg iBAT was further cut into four pieces. iBAT pieces were incubated in DMEM medium at 4 °C, and treated with or without OA (1 mM) for 4 h. p-(S/T)PKA substrate and GAPDH levels were measured by immunoblotting, and quantified by ImageJ.

### Generation of *Gpr3* KO mice

To generate *Gpr3* complete KO mice rapidly, we performed one-step complete knockout based on CRISPR/Cas9 systems, which was described previously.^68^ Briefly, four adjacent single-guide RNAs (sgRNAs) (spaced 50–100 bp apart; sgRNA1: TCTACAGGTACCATGATGTG; sgRNA2: CAGTGTGAACGTGAGCAGCG; sgRNA3: GGTGTCCTGCGAGAACGCGC; sgRNA4: ACCCACCAGCAGGAACATGG) that target key exon of *Gpr3* were designed. Subsequently, these four sgRNAs and Cas9 protein were microinjected into 174 zygotes of C57BL/6N mice, and 154 embryos that developed to 2-cell stage were transferred into 4 surrogate mothers. Two surrogates were successfully impregnated, and gave birth to 8 infants totally. By PCR genotyping, *Gpr3* of all F_0_ mice was completely deleted.

### Data analysis

Cell-based luciferase reporter assay data were analyzed in Microsoft Excel; cAMP assay and animal study data were analyzed in GraphPad Prism (version 7.1). For reporter assay, cAMP assay and animal study, data are represented as mean ± SD or SEM. Dots in the graph show individual data of three independent experiments, each performed in triplicate. Two-sided Student’s *t*-tests were used to compare the differences between two groups. The differences between three groups were examined using one-way analysis of variance (ANOVA) and the least significance difference (LSD) *t*-test. *P* < 0.05 is considered statistically significant. *P* values are indicated in the figure legends.

### Supplementary information

Supplementary information, Fig. S1

Supplementary information, Fig. S2

Supplementary information, Fig. S3

Supplementary information, Fig. S4

Supplementary information, Fig. S5

Supplementary information, Fig. S6

Supplementary information, Fig. S7

Supplementary information, Fig. S8

Supplementary information, Table S1

Supplementary information, Table S2

Supplementary information, Table S3

Supplementary information, Video S1

Supplementary Video S1 legend

## Data Availability

The cryo-EM density maps and atomic coordinates have been deposited in the Electron Microscopy Data Bank (EMDB) and Protein Data Bank (PDB) under accession numbers EMD-37881 and 8WW2, respectively, for the OA-bound GPR3/G_s_ complex.
