# Supplementary material for: Identification of oleic acid as an endogenous ligand of GPR3
Source: Cell Res. 2024 Jan 29;34(3):232–44. doi: 10.1038/s41422-024-00932-5 (PMC10907358; doi:10.1038/s41422-024-00932-5)
Supplement: Supplementary file 1 — Supplementary information, Fig. S1 [file 41422_2024_932_MOESM1_ESM.pdf]

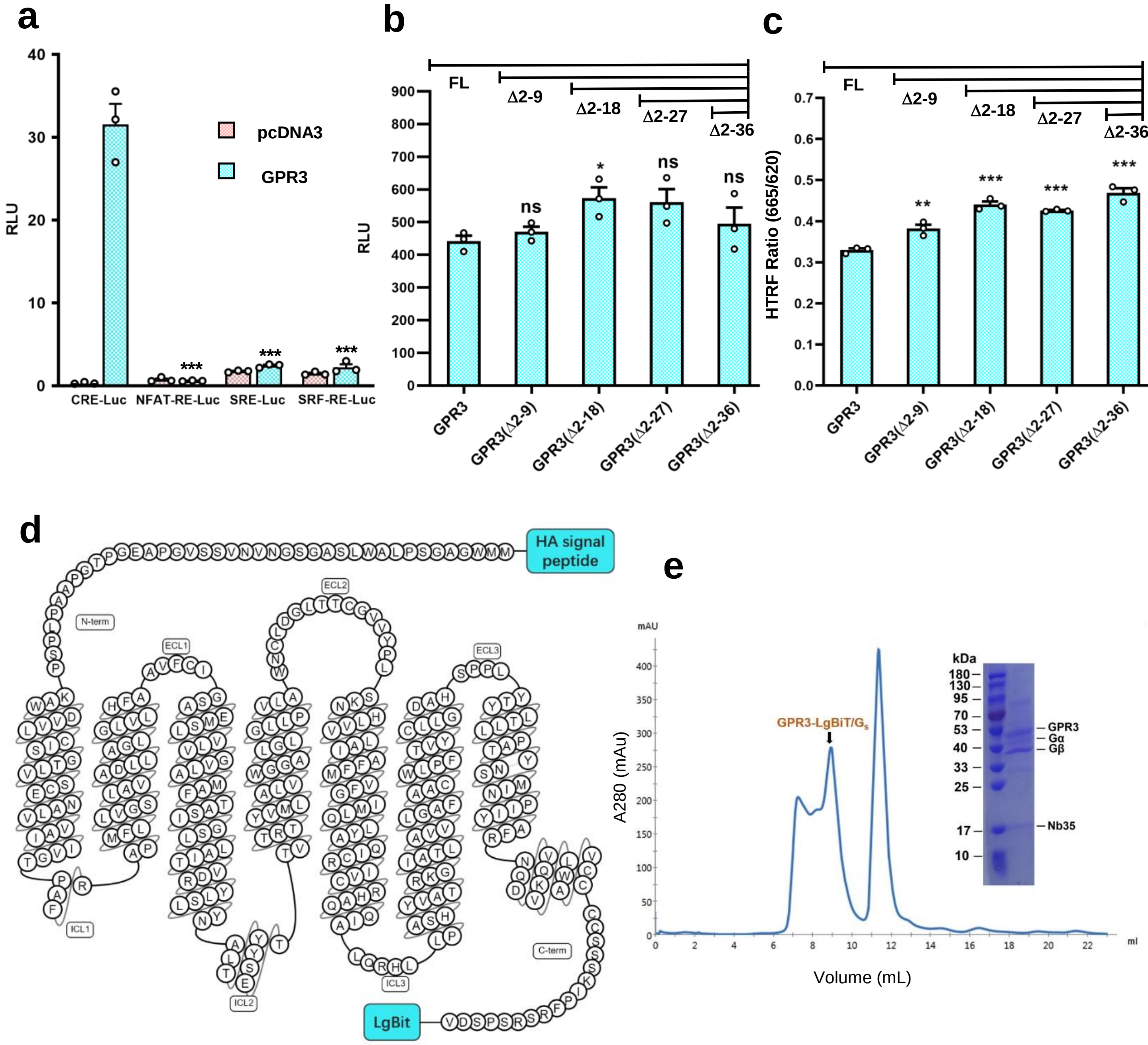

Supplementary information, Fig. S1. Constitutive activity of GPR3 and expression/purification of GPR3/G$_s$ complex. a An examination of the intrinsic activity of GPR3 via reporter assay. RLU, relative luciferase unit. Data are presented as mean values ± S.E.M.; n =3 independent samples; n.s. no significant; *, p < 0.05; **, p < 0.01; ***, p < 0.001. **b** An examination of N-terminal deletions of GPR3 in a CRE reporter assay. Data are presented as mean values ± S.E.M.; n =3 independent samples; n.s. no significant; *, p < 0.05; **, p < 0.01; ***, p < 0.001. **c** An examination of N-terminal deletion of GPR3 in a cAMP assay. Data are presented as mean values ± S.E.M.; n =3 independent samples; n.s. no significant; *, p < 0.05; **, p < 0.01; ***, p < 0.001. **d** A snake-shape diagram of human GPR3 construct used in complex assembling, the diagram was adopted from GPCRdb. **e** Size exclusion column profile of GPR3/G$_s$ complex.
