# Supplementary material for: Identification of oleic acid as an endogenous ligand of GPR3
Source: Cell Res. 2024 Jan 29;34(3):232–44. doi: 10.1038/s41422-024-00932-5 (PMC10907358; doi:10.1038/s41422-024-00932-5)
Supplement: Supplementary file 2 — Supplementary information, Fig. S2 [file 41422_2024_932_MOESM2_ESM.pdf]

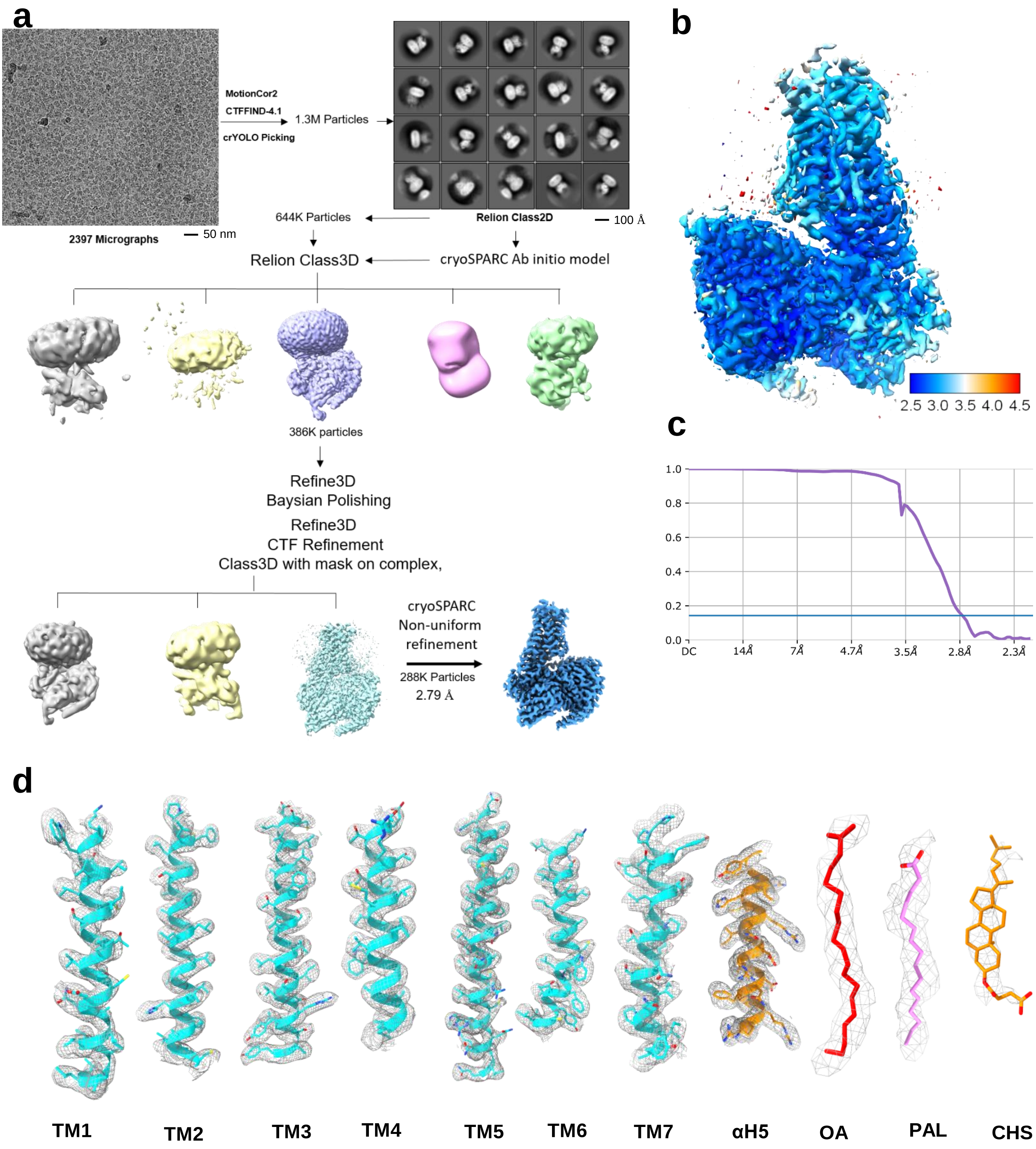

Supplementary information, Fig. S2. Single particle cryo-EM analysis of GPR3/G$_s$ complex. a Flow-chart of cryo-EM data process of GPR3/G$_s$ complexes. **b** Local resolution analysis of GPR3/G$_s$ complex. **c** FSC curve of the GPR3/G$_s$ complex. The resolution was assessed by the Gold Standard of FSC=0.143. **d** Cryo-EM density map of representative regions of GPR3/G$_s$ complex. Map level was set to 0.08 in ChimeraX.
