# Supplementary material for: Identification of oleic acid as an endogenous ligand of GPR3
Source: Cell Res. 2024 Jan 29;34(3):232–44. doi: 10.1038/s41422-024-00932-5 (PMC10907358; doi:10.1038/s41422-024-00932-5)
Supplement: Supplementary file 3 — Supplementary information, Fig. S3 [file 41422_2024_932_MOESM3_ESM.pdf]

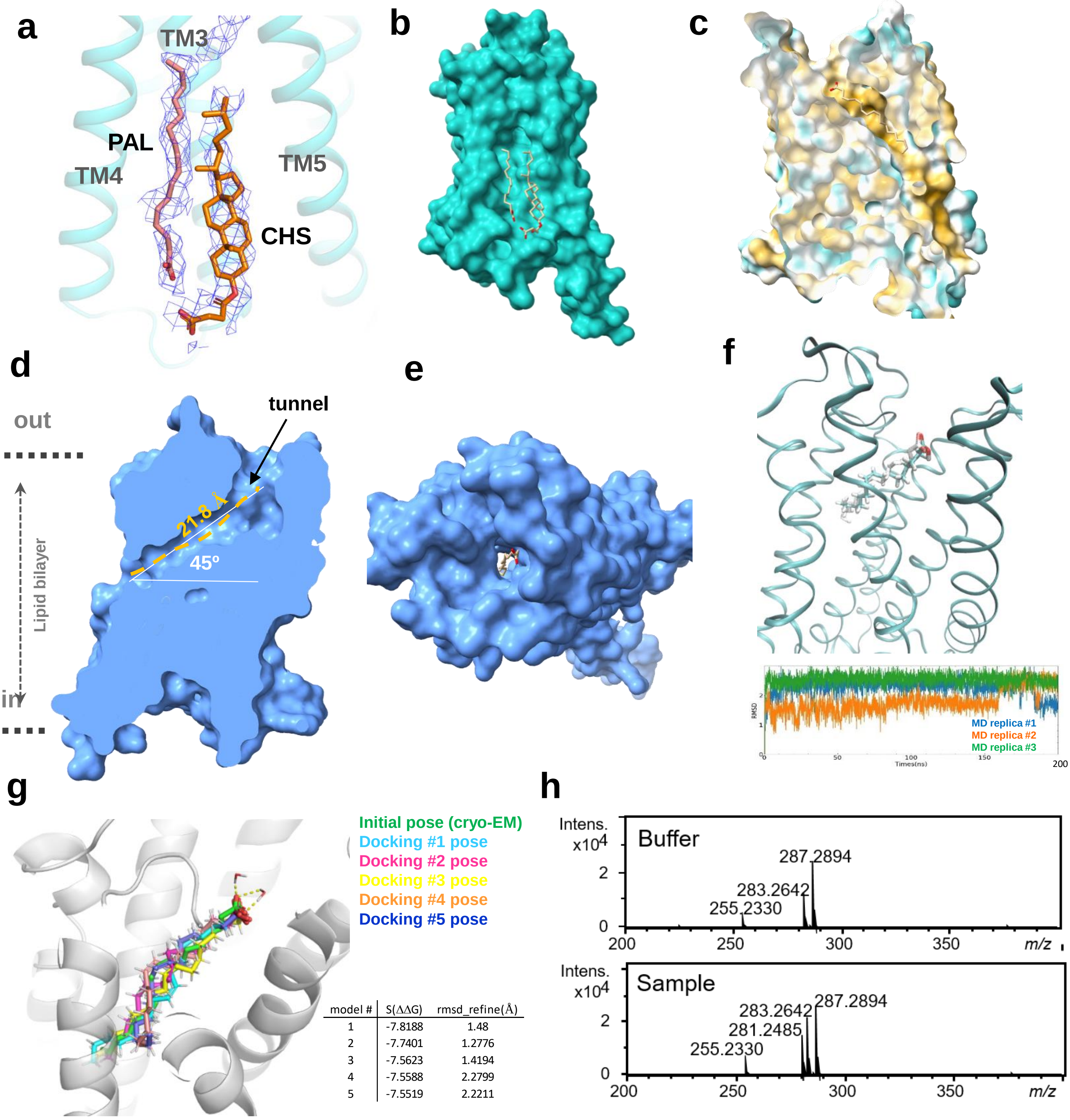

model #	S(ΔΔG)	rmsd_refine(Å)
1	-7.8188	1.48
2	-7.7401	1.2776
3	-7.5623	1.4194
4	-7.5588	2.2799
5	-7.5519	2.2211

Supplementary information, Fig. S3. Additional information of the ligand binding pocket of GPR3. a Density map of PAL (palmitic acid), and CHS (cholesterol hemisuccinate). Density map is set at contour level of 3.0 in pymol. **b** The position of PAL and CHS on a surface view of GPR3. **c** Hydrophobicity analysis of the ligand binding pocket of GPR3. **d** A hydrophobic tunnel penetrates receptor at an angle of 45º. **e** A top view of the channel from the extracellular side. **f** MD simulations of GPR3. Up panel, A snapshot of the simulation. The white color, OA position in the cryo-EM structure. Lower panel, RMSD analysis of OA position. **g** Docking analysis of OA binding in GPR3. **h** m/z plots of sample and buffer.
