# Supplementary material for: Identification of oleic acid as an endogenous ligand of GPR3
Source: Cell Res. 2024 Jan 29;34(3):232–44. doi: 10.1038/s41422-024-00932-5 (PMC10907358; doi:10.1038/s41422-024-00932-5)
Supplement: Supplementary file 4 — Supplementary information, Fig. S4 [file 41422_2024_932_MOESM4_ESM.pdf]

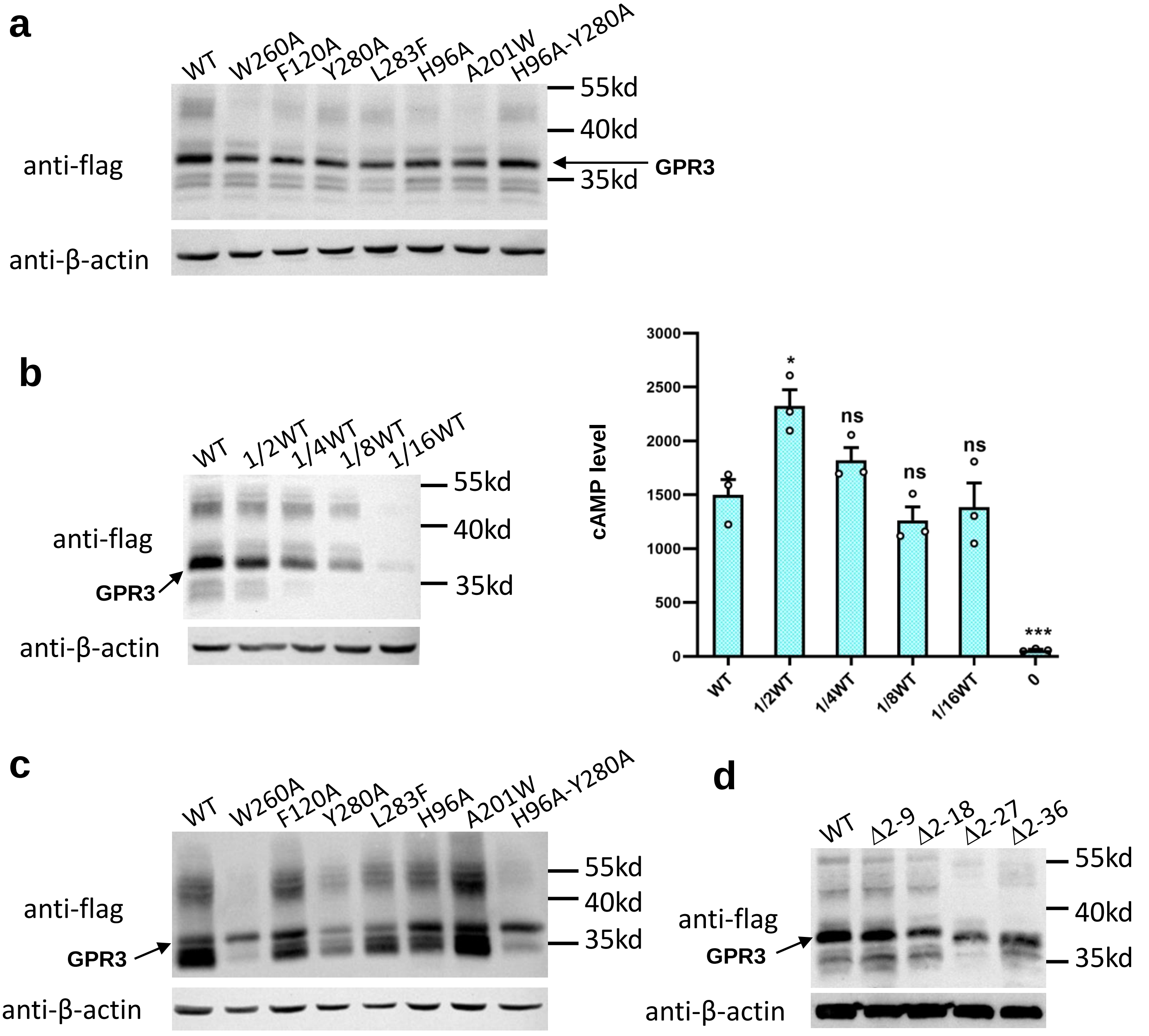

Supplementary information, Fig. S4. Expression of GPR3 mutants and the titration experiment. a Expression of GPR3 mutants. **b** The titration experiment of different amount of WT plasmids in the cAMP assay. ½ WT means transfection of ½ amount of WT GPR3 plasmid, ¼ means transfection of ¼ amount of WT GPR3 plasmid, and so on. **c** The protein expression level of GPR3 in the stably expressed GPR3 mutant colonies. **d** Expression of GPR3 N-terminal truncations.
