# Supplementary material for: Identification of oleic acid as an endogenous ligand of GPR3
Source: Cell Res. 2024 Jan 29;34(3):232–44. doi: 10.1038/s41422-024-00932-5 (PMC10907358; doi:10.1038/s41422-024-00932-5)
Supplement: Supplementary file 5 — Supplementary information, Fig. S5 [file 41422_2024_932_MOESM5_ESM.pdf]

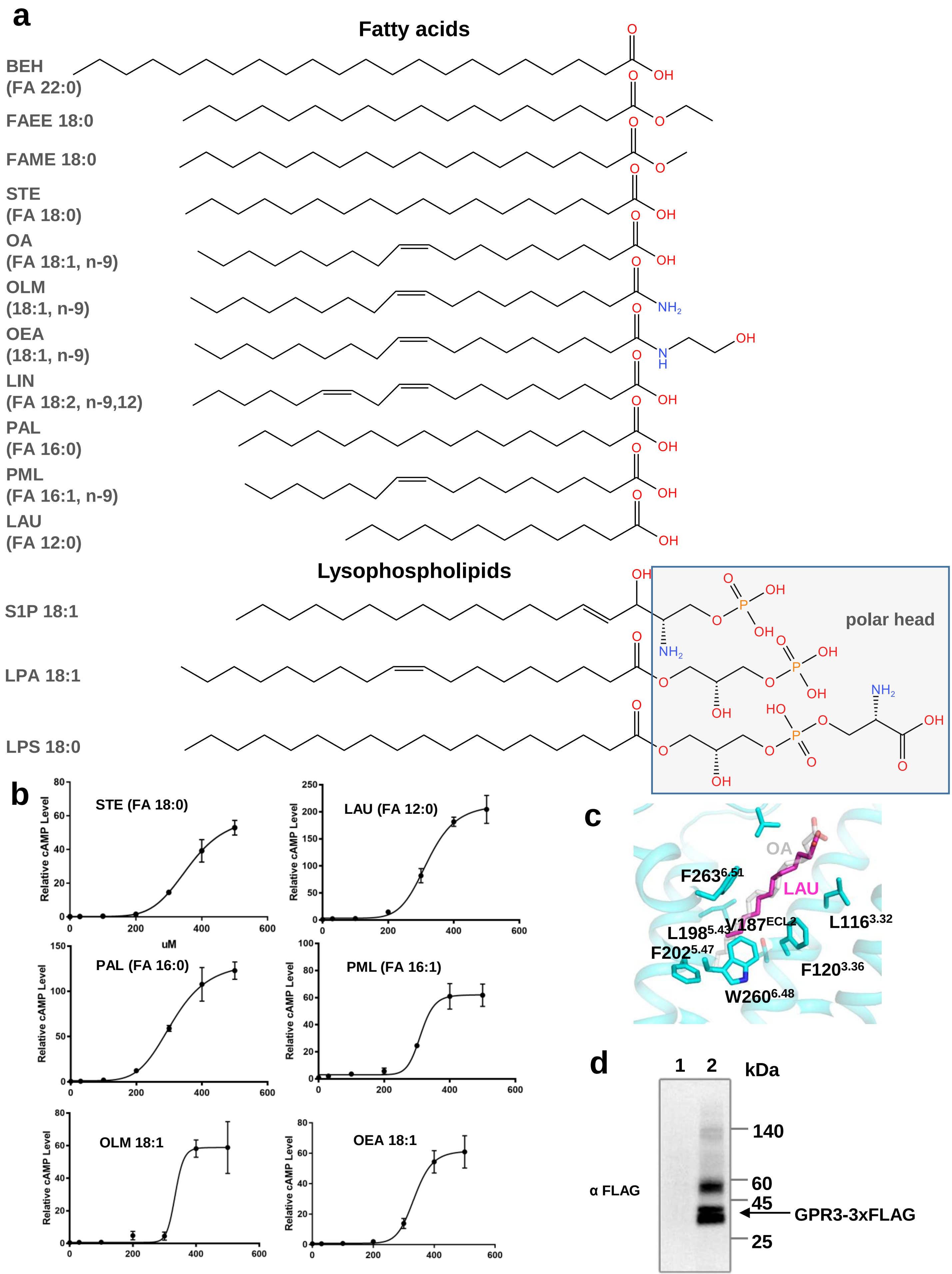
a
Fatty acids
BEH (FA 22:0)
FAEE 18:0
FAME 18:0
STE (FA 18:0)
OA (FA 18:1, n-9)
OLM (18:1, n-9)
OEA (18:1, n-9)
LIN (FA 18:2, n-9,12)
PAL (FA 16:0)
PML (FA 16:1, n-9)
LAU (FA 12:0)
Lysophospholipids
S1P 18:1
LPA 18:1
LPS 18:0
polar head
b
STE (FA 18:0)
LAU (FA 12:0)
PAL (FA 16:0)
PML (FA 16:1)
OLM 18:1
OEA 18:1
Relative cAMP Level
uM
c
OA
LAU
F263 6.51
L198 5.43
V187 ECL2
L116 3.32
F202 5.47
F120 3.36
W260 6.48
d
1
2
kDa
140
60
45
25
α FLAG
GPR3-3xFLAG

Supplementary information, Fig. S5. Additional information of GPR3 ligands. a Chemical structure of lipids in the study. BEH, behenic acid; FAEE 18:0, ethyl stearate; FAME 18:0, methyl stearate; STE, stearic acid; OA, oleic acid; LIN, linoleic acid; PAL, palmitic acid; PML, palmitoleic acid; LAU, lauric acid; S1P, sphigosine-1-phosphate; LPA, lysophosphatidic acid; LPS, lysophosphatidylserine. **b** Dose responses of additional lipids in GPR3 stably expressed AD293 cells. Data are presented as mean values ± S.E.M.; n =3 independent samples. The R value for curve fit of STE, LAU, PAL, PML, OLM and OEA are 0.9572, 0.962, 0.9499, 0.9304, 0.883 and 0.924, respectively. **c** A docking of lauric acid into GPR3 ligand binding pocket . **d** Western-blot of GPR3 stably expressed cell line. 1, lysate of AD293 cell line; 2, lysate of GPR3 stably expressed cell line.
