# Supplementary material for: Identification of oleic acid as an endogenous ligand of GPR3
Source: Cell Res. 2024 Jan 29;34(3):232–44. doi: 10.1038/s41422-024-00932-5 (PMC10907358; doi:10.1038/s41422-024-00932-5)
Supplement: Supplementary file 6 — Supplementary information, Fig. S6 [file 41422_2024_932_MOESM6_ESM.pdf]

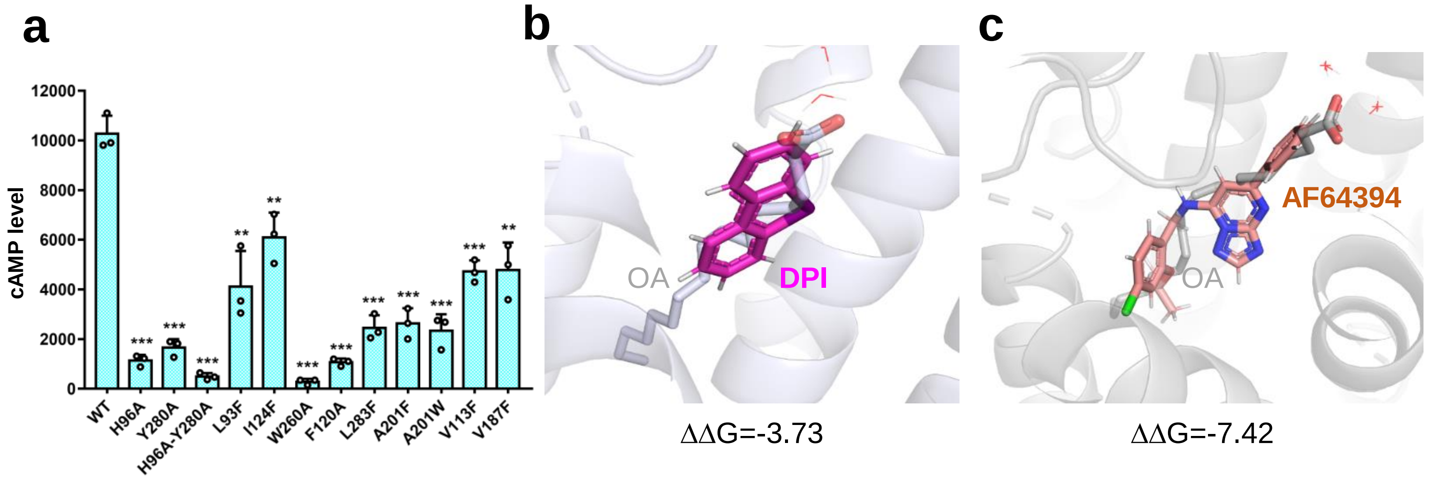

d

GPR3	L93$^{2.57}$	H96$^{2.60}$	V113$^{3.29}$	F120$^{3.36}$	V187^{ECL2}	A201$^{5.46}$	W260$^{6.48}$	Y280$^{7.36}$
GPR3	L	H	V	F	V	A	W	Y
GPR6	L	H	V	F	V	A	W	Y
GPR12	L	N	I	F	V	S	W	Y

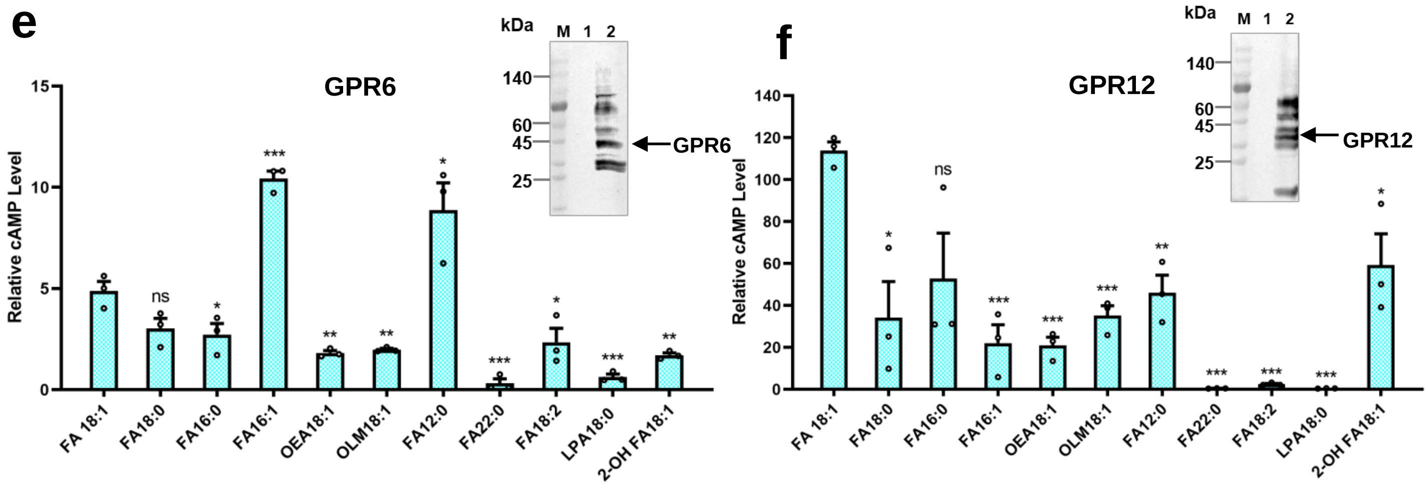

Supplementary information, Fig. S6. More additional information of GPR3 ligands. a A cAMP assay of GPR3 mutants under condition of without adding exogenous ligand (endogenous ligand of the medium). Data are presented as mean values ± S.E.M; n =3 independent samples; n.s. no significant; *, $P < 0.05$; **, $P < 0.01$; ***, $P < 0.001$. Two-side T-test. **b-c** Molecular docking of DPI and AF64394 into the ligand binding pocket of GPR3. **d** A sequence alignment of the key residues of the ligand binding pocket of GPR3, 6 and 12. **e-f** Activities of different lipids in a GRP6 (**e**) and GPR12 (**f**) stably expressed AD293 cell line via the GloSensor cAMP assay. Data are presented as mean values ± S.E.M; n =3 independent samples; n.s. no significant; *, $P < 0.05$; **, $P < 0.01$; ***, $P < 0.001$. Two-side T-test. Lipid concentration, 300 μM. Western-blot of GPR6 and GPR12 stably expressed cell line. 1, lysate of AD293 cell line; 2, lysate of GPR6 or GPR12 stably expressed cell line. Anti-FLAG.
