# Supplementary material for: Identification of oleic acid as an endogenous ligand of GPR3
Source: Cell Res. 2024 Jan 29;34(3):232–44. doi: 10.1038/s41422-024-00932-5 (PMC10907358; doi:10.1038/s41422-024-00932-5)
Supplement: Supplementary file 7 — Supplementary information, Fig. S7 [file 41422_2024_932_MOESM7_ESM.pdf]

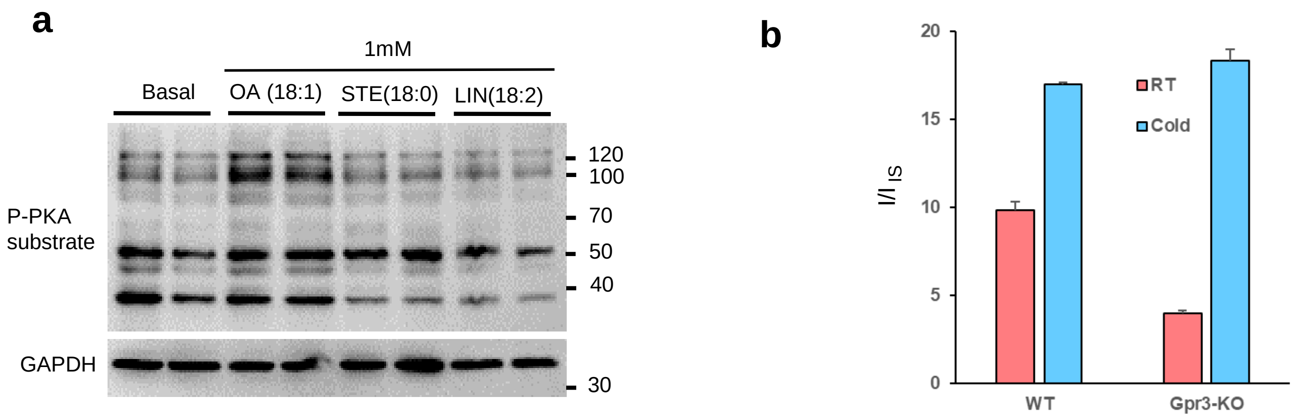

Supplementary information, Fig. S7. Additional data of cold-induced thermogenesis. a Examination of the effect of different lipids in inducing G_s/cAMP/PKA signaling under cold stimulation (4ºC) in WT iBAT. **b** LC/MS analysis of OA level of WT or Gpr3-KO mice upon cold stimulation. Data are presented as mean values ± SD; n=2.
