# Supplementary material for: Identification of oleic acid as an endogenous ligand of GPR3
Source: Cell Res. 2024 Jan 29;34(3):232–44. doi: 10.1038/s41422-024-00932-5 (PMC10907358; doi:10.1038/s41422-024-00932-5)
Supplement: Supplementary file 8 — Supplementary information, Fig. S8 [file 41422_2024_932_MOESM8_ESM.pdf]

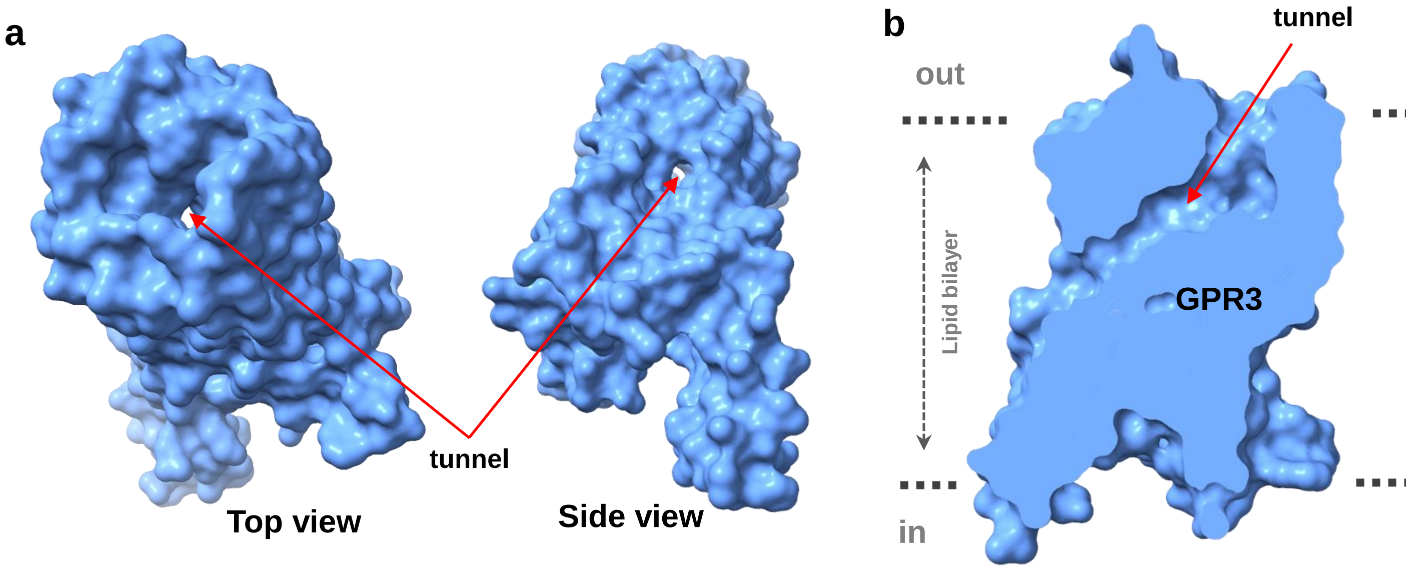

Supplementary information, Fig. S8. The lipid tunnel of GPR3. **a** The lipid tunnel of apo GPR3 (AF-P46095).
b The transverse cross section of the lipid tunnel of GPR3.
