# Supplementary material for: Identification of oleic acid as an endogenous ligand of GPR3
Source: Cell Res. 2024 Jan 29;34(3):232–44. doi: 10.1038/s41422-024-00932-5 (PMC10907358; doi:10.1038/s41422-024-00932-5)
Supplement: Supplementary file 9 — Supplementary information, Table S1 [file 41422_2024_932_MOESM9_ESM.pdf]

Supplementary information, Table S1 Cryo-EM data collection and refinement statistics

	GPR3/OA/G$\alpha_s\beta\gamma$/Nb35<br>EMD-37881<br>8WW2
**Data collection and processing**	
Magnification	130,000
Voltage (kV)	300
Electron exposure ($e^-/Å^2$)	60
Defocus range (μm)	1.2-2.2
Pixel size (Å)	0.55
Symmetry imposed	C1
Initial particle image (no.)	1.3M
Final particle image (no.)	288k
Map resolution (Å)	2.79
FSC threshold	0.143
**Refinement**	
Initial model used (PDB code)	alpha-fold (AF-P46089), 6vn7
Model Resolution (Å)	3.3
FSC threshold	0.143
Map sharpening *B* factor ($Å^2$)	-115.4
Model composition	
Non-hydrogen atoms	7934
Protein residues	1021
Ligands	3
B factor ($Å^2$)	
Protein	44.01
Ligand	56.44
R.m.s. deviations	
Bond length (Å)	0.006
Bond angles (°)	0.818
Validation	
MolProbity score	1.55
Clashscore	4.81
Poor rotamers (%)	0
Ramachandran plot	
Favored (%)	95.72
Allowed (%)	4.28
Disallowed	0
