# Supplementary material for: Identification of oleic acid as an endogenous ligand of GPR3
Source: Cell Res. 2024 Jan 29;34(3):232–44. doi: 10.1038/s41422-024-00932-5 (PMC10907358; doi:10.1038/s41422-024-00932-5)
Supplement: Supplementary file 10 — Supplementary information, Table S2 [file 41422_2024_932_MOESM10_ESM.pdf]

Supplementary information, Table S2 lipids activities on GPR3

ligand	FA 18:1	FA 18:0	FA 16:0	FA 16:1	OEA 18:1	OLM 18:1	FA 12:0	FA 22:0	FA 18:2	FAME 18:0
maximum response	164.1	44.8	118.7	61.8	60.9	75.1	204.5	1.0	2.9	0.9
Ec50 (μM)	298.9	361.2	311.0	328.4	335.9	333.9	320.1	N/A	N/A	N/A

ligand	FAEE 18:0	LPA 16:0	LPA 17:0	LPA 18:0	LPA 18:1	LPS 18:0	LPC 14:0	LPC 16:0	S1P 18:1	LTB4
maximum response	0.6	0.5	0.5	0.6	0.5	0.6	1.8	0.7	0.6	0.8
Ec50 (μM)	N/A	N/A	N/A	N/A	N/A	N/A	N/A	N/A	N/A	N/A
