# Supplementary material for: Identification of oleic acid as an endogenous ligand of GPR3
Source: Cell Res. 2024 Jan 29;34(3):232–44. doi: 10.1038/s41422-024-00932-5 (PMC10907358; doi:10.1038/s41422-024-00932-5)
Supplement: Supplementary file 11 — Supplementary information, Table S3 [file 41422_2024_932_MOESM11_ESM.pdf]

Supplementary information, Table S3 OA activities on GPR3 mutants

Mutant	WT	W260A	F120A	Y280A	L283F	H96A	A201W	H96A-Y280A
maximum response	186.6	N/A	23.67	61.91	39.13	83.68	157.9	N/A
Ec50 (μM)	293.2	N/A	252.2	322.0	N/A	254.5	291.4	N/A
