# Supplementary material for: Identification of oleic acid as an endogenous ligand of GPR3
Source: Cell Res. 2024 Jan 29;34(3):232–44. doi: 10.1038/s41422-024-00932-5 (PMC10907358; doi:10.1038/s41422-024-00932-5)
Supplement: Supplementary file 13 — Supplementary Video S1 legend [file 41422_2024_932_MOESM13_ESM.pdf]

Supplementary information, Video S1 Movie track of MD simulation of OA-bound GPR3. The time length is 200 ns, interval is 5 steps.
